# Serum Amyloid A Stimulates Vascular and Renal Dysfunction in Apolipoprotein E-Deficient Mice Fed a Normal Chow Diet

**DOI:** 10.3389/fimmu.2019.00380

**Published:** 2019-03-07

**Authors:** Belal Chami, Farjaneh Hossain, Thomas W. Hambly, Xiaoping Cai, Roshanak Aran, Genevieve Fong, Abigail Vellajo, Nathan J.J Martin, XiaoSuo Wang, Joanne M. Dennis, Arpeeta Sharma, Waled A. Shihata, Jaye P. F. Chin-Dusting, Judy B. de Haan, Alexandra Sharland, Carolyn L. Geczy, Ben Freedman, Paul K. Witting

**Affiliations:** ^1^Discipline of Pathology, Charles Perkins Centre, The University of Sydney, Sydney, NSW, Australia; ^2^Department of Medicine, Monash University, Melbourne, VIC, Australia; ^3^Cardiovascular Disease Program, Biomedicine Discovery Institute, Monash University, Melbourne, VIC, Australia; ^4^Department of Pharmacology, Monash University, Melbourne, VIC, Australia; ^5^Baker Heart and Diabetes Institute, Melbourne, VIC, Australia; ^6^Transplantation Immunobiology Group, Charles Perkins Centre, The University of Sydney, Sydney, NSW, Australia; ^7^School of Medical Sciences, University of New South Wales, Sydney, NSW, Australia; ^8^ANZAC Research and Heart Research Institutes, Charles Perkins Centre, University of Sydney, Sydney, NSW, Australia

**Keywords:** endothelial function, inflammation, nitric oxide, acute-phase protein, renal dysfunction

## Abstract

Elevated serum amyloid A (SAA) levels may promote endothelial dysfunction, which is linked to cardiovascular and renal pathologies. We investigated the effect of SAA on vascular and renal function in apolipoprotein E-deficient (ApoE^−/−^) mice. Male ApoE^−/−^ mice received vehicle (control), low-level lipopolysaccharide (LPS), or recombinant human SAA by *i.p*. injection every third day for 2 weeks. Heart, aorta and kidney were harvested between 3 days and 18 weeks after treatment. SAA administration increased vascular cell adhesion molecule (VCAM)-1 expression and circulating monocyte chemotactic protein (MCP)-1 and decreased aortic cyclic guanosine monophosphate (cGMP), consistent with SAA inhibiting nitric oxide bioactivity. In addition, binding of labeled leukocytes to excised aorta increased as monitored using an *ex vivo* leukocyte adhesion assay. Renal injury was evident 4 weeks after commencement of SAA treatment, manifesting as increased plasma urea, urinary protein, oxidized lipids, urinary kidney injury molecule (KIM)-1 and multiple cytokines and chemokines in kidney tissue, relative to controls. Phosphorylation of nuclear-factor-kappa-beta (NFκB-*p*-P65), tissue factor (TF), and macrophage recruitment increased in kidneys from ApoE^−/−^ mice 4 weeks after SAA treatment, confirming that SAA elicited a pro-inflammatory and pro-thrombotic phenotype. These data indicate that SAA impairs endothelial and renal function in ApoE^−/−^ mice in the absence of a high-fat diet.

## Introduction

A functional endothelium is vital for maintenance of vascular homeostasis (1). Endothelial cell (EC) dysfunction, characterized by altered nitric oxide (^**•**^NO) production/bioactivity, precedes atherosclerosis (2), essential hypertension (3), and related cardiovascular disease (CVD). Endothelium-derived ^**•**^NO is produced *via* the action of endothelial nitric oxide synthase (eNOS) on *L*-arginine (*L*-Arg), regulates vascular tone (4), and interactions between platelets (5), leukocytes (6), and the endothelium as well as modulating vascular smooth muscle cell (VSMC) proliferation (7).

Serum amyloid A (SAA) is a family of acute phase apo-lipoproteins, principally produced by hepatocytes and macrophages. Circulating levels rise (up to 1,000-fold) in an acute phase response; TNF, IL-1, and IL-6 with endogenous glucocorticoids are co-stimulants (8). SAA levels are elevated in chronic vascular diseases (9) and predict CVD risk (10, 11). SAA associates with high-density lipoprotein (HDL), chiefly HDL_3_ (9). SAA gene transcription is mediated by SAA-activating sequence binding factor, NFκB, NF-IL-6, and Sp1 (12).

SAA is deposited in atherosclerotic lesions and produced by activated monocytes/macrophages, VSMC, and EC (13). We reported a positive transcoronary gradient of SAA in patients with acute coronary syndromes (14), confirming localized vascular production of this acute phase protein. Furthermore, SAA inhibits endothelium-dependent VSMC relaxation by increasing EC-derived superoxide radical anion generation *in vitro* (15), an activity inhibited by added HDL (16). Pro-inflammatory SAA induces IL-6, IL-8, and TNF in neutrophils and monocytes (17) and promotes monocyte chemotaxis and leukocyte adhesion (18, 19). Transgenic over-expression of human SAA stimulates pro-atherogenic events in the vessel wall of apolipoprotein E-deficient (ApoE^−/−^) mice (20), including proteoglycan synthesis and retention of low-density lipoprotein (LDL)-like particles, and accelerated lesion formation at the aortic root in these animals (20, 21). SAA mediates HDL binding to macrophages and EC (22), impairs HDL's ability to promote cholesterol efflux from macrophages (23), and binds vascular proteoglycans (24), processes critical for facilitating retention of pro-atherogenic lipoproteins within the vessel wall. SAA can displace Apo-AI from HDL (9) and when SAA concentrations are chronically elevated it dissociates from HDL, yielding SAA, lipid-poor ApoA-I, and lipoprotein-free SAA (25, 26). Whether HDL-associated SAA impairs the lipoprotein's anti-inflammatory action is unclear. One study reported no effect on vascular EC (27), whereas another indicated that uremic subjects displayed up to 49 HDL-associated proteins but only SAA activity correlated with the pro-inflammatory state associated with end-stage renal disease (28).

SAA is also implicated in renal damage during reactive systemic amyloidosis in patients with chronic inflammatory disorders presenting with nephropathy (29). Chronically elevated levels of circulating hepatic SAA form amyloid fibrils, which accumulate within glomeruli, causing irreversible structural damage. Despite this, little is known regarding effects of acute SAA exposure upon the renal vasculature.

Here we demonstrate that short-term administration of recombinant SAA to ApoE^−/−^ mice impaired renal function and induced pro-atherogenic factors on the vascular endothelium that potentially accelerate atherosclerosis in the absence of a high-fat diet.

## Materials and Methods

Biochemicals including lipopolysaccharide (LPS; *E. coli* 055:B5) were from Sigma (Sydney, Australia) unless otherwise stated. Solutions were freshly prepared using MilliQ® water or high-quality analytical grade solvents (ACS) and where appropriate, sterilized prior to use. Recombinant human SAA (PeproTech, Lonza, Mount Waverly, Australia) is a consensus molecule of the SAA1/2 isoforms and has been used in numerous *in vitro* studies (17–19). Reagents and media were rigorously tested for endotoxin levels using the Limulus Amebocyte Lysate (LAL) buffer and endotoxin standards, visualized with Spectrozyme LAL (American Diagnostica, Stamford, CA). Reagents were discarded if endotoxin levels were >5 pg/mL. We tested preparations of recombinant SAA for LPS contamination and routinely quantified <2 pg LPS/μg SAA/mL, an amount unable to elicit pro-inflammatory/pro-coagulant responses in human peripheral blood monocytes that are highly sensitive to LPS (14). Recombinant SAA activity is ablated by boiling (30) whereas LPS retains pro-inflammatory activity indicating that recombinant SAA itself can induce an inflammatory response in isolated immune cells.

### Animals

Male ApoE-deficient mice (ApoE^−/−^, 8–10 weeks of age, weight 20–22 g) were sourced from the Animal Resources Center (Perth, Western Australia), housed in groups of 3–5/cage and maintained (22°C, 12 h light-dark cycle) at the Heart Research Institute (designated as study group 1, Sydney, Australia) or the Baker Heart and Diabetes Institute (designated as study group 2, Melbourne, Australia). Mice were fed standard laboratory chow and water *ad libitum*. All procedures were approved by the Sydney South West Area Health Services Ethics Committee or the Alfred Medical Research and Education Precinct (AMREP) animal ethics committee (Melbourne, Australia) prior to conducting studies.

### Experimental Design

#### Study Group 1

Eight-week-old male ApoE^−/−^mice were randomly allocated into three groups (numbers of mice in each group as indicated in the legends to the figures): control (sterile saline as a vehicle), LPS (stock solution 100 pg/mL; final LPS dose 25 pg/kg), and an SAA group (stock solution 120 μg/mL in saline; final SAA dose 10 μg/kg) via i.p. injection. Note, a dose of LPS slightly higher than that estimated from the corresponding level of contamination in the final SAA dose administered to mice was selected as a second control to account for the possibility that the bioactivity activity of recombinant SAA was due to contaminating LPS. Mice were treated with saline, LPS or recombinant SAA from day 1, then every third day for a total of 14 days, then euthanized either 4 or 18 weeks after commencement of treatment. To assess infiltrating cells in the kidney at an early time point following SAA treatment, additional ApoE^−/−^ mice were randomly allocated into the three groups as above, given saline, LPS or SAA i.p, then euthanized 3 or 5 days after commencement of treatment. These time points were selected to mimic acute innate tissue response (3–5 days), general tissue inflammation (4 weeks after final SAA treatment) or a chronic tissue response (18 weeks after final SAA treatment).

#### Study Group 2

Eight-week-old male ApoE^−/−^ mice on a C57BL/6 background were ear-tagged and allowed to acclimatize prior to performing *ex vivo* leukocyte adhesion studies. Mice received SAA (10 μg/kg; i.p.) injection on days 8, 11, and 13, or sterilized PBS (vehicle control) on days 0, 8, 11, and 13 and were culled on day 14 for use in vascular adhesion studies. This short time period from final SAA treatment to conducting leukocyte adhesion studies was selected to optimize the vascular response to pro-inflammatory SAA. Leukocyte-endothelial interactions were observed using an *ex vivo* dynamic flow adhesion assay, as previously described (31). Fewer mice were employed for treatment groups in “Study group 2″ due to the complexity and labor-intensive nature of the experimental work conducted.

Abdominal aortae were excised, and each end of the abdominal aorta was then carefully mounted on the cannula in a vessel chamber. Freshly isolated human blood was labeled with VybrantDil dye (Invitrogen, USA; final dilution 1:1000 v/v, 10 min) and then perfused at a rate of 7.5 mL/h through the isolated aorta. Real-time leukocyte adhesion interactions were then visualized using a fluorescence microscope. Video clips (15 s duration) were acquired at 100*x* magnification with a field of view of 1.4 mm and readings taken in one field along the vessel at 5 separate time points during 2.5 min intervals. Adherent leukocytes were enumerated if they remained adherent to the vessel wall during each 15 s video recording. These experiments were performed at the Baker Institute and Monash University, Melbourne.

### Collection of Urine, Blood and Organs for Analyses

Prior to collection of blood and organs, mice were restrained by grasping the scruff of the neck and urine samples from individual male mice were collected directly into screw cap tubes and frozen immediately in liquid nitrogen for subsequent biochemical analyses. Under these conditions mice typically yielded 10–50 μL urine. Mice were then anesthetized with isofluorane, a mid-line incision made in the abdomen and a thoracotomy performed to expose the heart. Whole blood (0.5–1 mL) was collected by direct left ventricle puncture into a syringe containing heparin (100 IU), centrifuged (680 × g), and plasma removed and stored at −80°C for subsequent analyses.

Following cardiac puncture, the vasculature was perfused with PBS (80 mm Hg) and organs resected. The heart and left kidney were assigned to histology and fixed in 4% v/v paraformaldehyde and subsequently transferred to 70% v/v ethanol. The right kidney was sectioned longitudinally, and one half immersed in buffer [50 mM phosphate buffer, pH 7.4 containing: 1 mM EDTA, 10 μM butylated-hydroxytoluene, 1 protease inhibitor cocktail tablet/50 mL (Roche, Switzerland)] and frozen in liquid nitrogen. This sample was used to prepare homogenates and subsequent mRNA, protein, or lipid analysis. The other half of the right kidney was frozen in cryopreservative (Tissue-Tek OCT, Sakura Finetek, The Netherlands) and stored at −80°C until used for immunohistochemistry.

### Kidney Morphology

Renal morphology was examined using formaldehyde-fixed, paraffin embedded sections. To assess morphological changes, kidney sections were stained with periodic acid Schiff (PAS); Congo red stain was used to assess renal amyloidosis (32). Imaged sections were viewed and captured using an Olympus Photo Microscope fitted with a digital camera (DP Controller; *v2.2.1.227*). Images were converted to JPEG and collated using Microsoft Powerpoint (*v7*). Image analysis software, Image-pro Plus (*v6*) was used to delineate the region defined by Bowman's space to determine the encapsulated area as a percentage of the total area defined by Bowman's capsule.

### Immunohistochemistry

#### Preparation of Fixed Tissues and Antigen Retrieval

De-paraffinised sections were dewaxed, rehydrated and cycled through an antigen retrieval process either by boiling in citrate buffer (10 mM, pH 6.0) or Tris/EDTA buffer (10 mM, pH 9.0) for 10 min. Endogenous peroxidase activity and non-specific immunoreactivity was blocked by incubating in 3% v/v methanolic H_2_O_2_ and 5% v/v goat serum/PBS for 30 min, respectively.

Experimental conditions for use of primary and secondary antibodies are summarized in Supplementary Table 1. Paraformaldehyde-fixed aortae were sectioned (5 μm) and incubated with anti-mouse glutathione peroxidase-1 (kidney; Abcam, Sydney, Australia), monoclonal anti-VCAM-1 (aorta; Santa Cruz, Sapphire Biosciences, Sydney, Australia), anti-Ly6G (kidney; Abcam, Melbourne, Australia), or a fluorescein isothiocyanate (FITC)-conjugated monoclonal anti-TF antibody (kidney; American Diagnostic Inc, Stamford, CT) in a humidified chamber (2 h, 20°C) (as detailed in Supplementary Table 1). Control sections were incubated with either PBS (negative control) or appropriate rabbit/mouse IgG (isotype controls). Sections were then washed, incubated with biotinylated secondary antibody (either goat anti-rabbit or anti-mouse IgG, for 1 h in a humidified chamber), then for 30 min with a Streptavidin-HRP reagent (VECTASTAIN® ABC kit; Vector Laboratories, CA) followed by reaction with diaminobenzidine (DAB; DAKO Cytomation, CA) as visualizing agent. Sections were counterstained with Harris's hematoxylin or imaged directly with a BX60 microscope (Olympus Australia, Notting Hill, Victoria) fitted with a fluorescence source.

#### Preparation of Frozen Tissue

For identification of neutrophils and macrophages, kidney samples frozen in Tissue-Tek OCT were carefully warmed at 22°C, relocated into cryomolds without the entire specimen thawing (ProSciTech, Thuringowa, Queensland, Australia) and refrozen in liquid nitrogen before transfer to a cryotome. Renal tissues were sectioned (7 μm), air dried, fixed in ice-cold acetone and rinsed with distilled water. Endogenous peroxidases were quenched as described above and treated sections subsequently incubated with an Fc receptor blocker (Innovex, Richmond, USA), 1:3 (v/v) in 1% (w/v) BSA and 0.5% (v/v) Triton X-100 in Tris-buffered saline (TBST; 30 min) then washed (3x TBST,1 min).

#### Myeloperoxidase (MPO) and Macrophage Co-labeling in Mouse Kidney

Renal sections were incubated with rat anti-mouse F4/80 antibody (1 h, 20°C), washed in TBST (1 min) then treated with an HRP-conjugated goat anti-rat IgG antibody (Merck Millipore, Kilsyth, Australia) for 30 min. Slides were then washed with TBST (1 min) and the developed (20°C, 6 min) with DAB substrate (Dako, North Sydney, Australia).

Sections were then stained for MPO, a heme enzyme present in neutrophils and macrophages. Mounted renal specimens were incubated with rabbit polyclonal anti-MPO antibody (1 h), washed in TBST and incubated with FITC-conjugated goat anti-rabbit IgG (Abcam Cat#ab97050), in a humid environment (30 min, 20°C). Slides were then washed with TBST (4*x* 5 min) and dipped in Sudan Black B solution (1% v/v, 3 min) to reduce background autofluorescence.

Slides were left in TBST wash solution overnight before counterstaining with DAPI. Images were digitally processed with Photoshop CS2 software (Adobe Systems, Sydney, Australia). DAB-stained images were processed to mimic red immunofluorescence, by digitally subtracting the blue color channel from the red color channel using the Apply Image tool. The processed color channel was then superimposed over DAPI and FITC channels to form the final image as described previously (33).

#### NFκB and Macrophage Co-expression

Mounted tissue sections were incubated with an antibody cocktail consisting of a rat anti-mouse F4/80 antibody and a rabbit anti-mouse NFκB p65 phospho-Ser276 antibody (Signalway, Australian BioBest Biotechnology, Sydney) in 1% w/v BSA diluent, for 1 h. Sections washed in TBST were incubated with a cocktail consisting of Alexa Fluor 594 conjugated donkey anti-rat IgG (Life Technologies, Mulgrave, Australia) and a FITC conjugated goat anti-rabbit IgG (Abcam, Waterloo, Australia), in BSA diluent (1% w/v, 30 min). Slides were then washed with TBST and incubated for Sudan black B solution (1% w/v, 3 min) to reduce autofluorescence. Following washes in TBST (3*x* 5 min), sections were counterstained with SlowFade Gold (Invitrogen, Thermo Fisher Scientific Australia, Scoresby, Australia; cat#S36938) fluorescence mounting medium containing 4′,6-diamidino-2-phenylindole (DAPI) before cover-slipping. Representative images were taken with a DP71 camera (Olympus Australia, Notting Hill, Australia) and documented with CellSens Standard software (v1.6, Olympus Australia, Notting Hill, Australia).

### Assessing Markers of Renal Injury

Total renal homogenate protein was measured using the bicinchoninic acid (BCA) assay (Sigma-Aldrich, Sydney Australia) with an albumin standard (Sigma-Aldrich, Sydney Australia) as described previously (34). Urinary protein was determined using a Direct Detect® method with infra-red based spectrometry that directly quantifies amide bonds and is not reliant on binding of dyes to amino acids in proteins (Cat# DDHW00010-WW; Merck-Millipore, Sydney Australia). Urinary kidney injury molecule-1 (KIM-1) was determined with an ELISA kit (ADIPO Bioscience, Santa Clara, CA) following the manufacturer's instructions and commercial standards provided in the kit.

### CCL-2/MCP-1 Assessment

Pro-inflammatory chemokine MCP-1 concentrations in plasma were quantified using an AlphaLISA® CCL-2/MCP-1 kit, following the manufacturer's instructions, and absorbance determined on a Pherastar Microplate Reader (BMG Labtech, Ortenberg, Germany).

### Plasma Urea

Plasma was diluted 1:10 v/v in PBS (50 mM, pH 7.4) and an aliquot (50 μL) incubated (90 min, 37°C) in carbonate buffer (100 mM, pH 9) containing 10 mM MnCl_2_ (50 μL), then 100 μM 2,3-butanedione monoxime solution was added and the mixture incubated (2 h, 37°C) as we described previously (16). Urea levels were determined by monitoring the time-dependent increases in absorbance at A_515__nm_ using a microplate reader (BioRad, Gladesville, Sydney, Australia).

### Preparation of Renal and Aortic Tissue Homogenates

Tissue homogenates were prepared with a rotating piston and matching Teflon-coated tube as described previously (35, 36). Briefly, frozen kidneys or aortae were thawed, diced, snap frozen in liquid nitrogen, and pulverized to a powder with a mortar and pestle. The powdered tissue was suspended in buffer [50 mM PBS, pH 7.4 containing: 1 mM EDTA, 10 μM butylated hydroxytoluene, and a Protease Inhibitor Cocktail tablet (Roche Diagnostics, Bern Switzerland)] then transferred to a glass tube and homogenized on ice with a matching rotating piston (Wheaton Specialty Glass, USA; at 500 r.p.m.). After 5 min, a sample (50 μL) was taken for protein analysis and the remainder split into two equal volumes. One sample was stored at −80°C for mRNA extraction, cytokine, and biochemical analyses, the other immediately placed into a mixture of hexane and methanol (5:1 v/v) for lipid extraction. The lipid-soluble hexane fraction was separated, dried under reduced pressure, resuspended in isopropanol (200 μL) then lipid analysis conducted by liquid chromatography. Samples assigned to gene analysis were thawed, centrifuged (360 × g, 5 min), supernatants removed then re-centrifuged (360 × g, 5 min) to remove solid debris (referred to as clarified homogenate). The clarified homogenate was used for mRNA extraction and/or biochemical analyses (below). As the availability of renal/aortic homogenate became limiting, due to the large number of biochemical assessments conducted in this study, sample number in each treatment group varied from 10 to 4 in some assays; however, this did not preclude suitable statistical comparisons between groups albeit that the data sets were not normally distributed and required the use of non-parametric statistical tests.

### Renal and Aortic Biochemistry

Total glutathione peroxidase (GPx) activity was determined in clarified kidney homogenates as described (36). Briefly, two buffers were freshly prepared: (i) Mastermix A (50 mM PBS; pH 7.4 containing: 1 mM EDTA, 1 mM sodium azide, 1 mM glutathione GSH, 1 U glutathione disulfide (GSSG) reductase, and 0.25 mM NADPH) and (ii) Mastermix B (50 mM PBS; pH 7.4 containing: H_2_O_2_, final concentration 350 mM). Initially, clarified tissue homogenates (10 μL) were diluted with 90 μL Mastermix A in a 96-well plate then 65 μL Mastermix B added and the mixture thoroughly mixed and incubated at 20°C. Time-dependent changes in absorbance were then monitored at A340 nm at 0, 2, 4, and 6 min. Total GPx activity was defined as the maximal rate of NADPH consumption expressed as a protein-normalized fold-change relative to the control group.

Total arginase activity was determined in clarified aortic tissue homogenates that were heat activated (90 min, 37°C) in carbonate buffer (100 mM, pH 9) containing 10 mM MnCl_2_ then mixed with 250 mM *L*-Arg. Samples were incubated at 37°C with a 100 μM 2,3-butanedione monoxime (Sigma-Aldrich, Sydney, Australia) and urea production monitored (A_515nm_, every 15 min over 4 h); arginase activity was expressed as pmol urea/unit time/mg protein.

Cyclic guanosine monophosphate (cGMP) is produced by the activation of soluble guanyly cyclase upon activation of bioactive NO. The concentration of this secondary messenger was determined in aortic homogenates with an ELISA kit (Sapphire Biosciences, Redfern, Sydney, Australia) as previously described (37). Aortic cGMP was then determined in the clarified homogenates and quantified against standard curves generated on the same ELISA plate and normalized to total homogenate protein in the corresponding sample.

### Renal Tissue and Plasma Lipid Analysis

The lipid-soluble components from clarified renal tissue homogenates or plasma, including α-tocopherol (referred to as α-TOH), free cholesterol (FC), cholesteryl esters [cholesteryl linoleate (C18:2) and cholesteryl arachidonic (C20:4) (together referred to as CE)], and CE-derived lipid hydroperoxides and hydroxides [together referred to as CEO(O)H], were measured by high performance liquid chromatography with peak validation and quantitation using authentic standards as described previously (35, 36, 38).

Renal tissue and plasma levels of F_2_-isoprostanes (biomarker of free radical-mediated arachidonic acid oxidation) were determined by immunoassay (Cayman, Ann Arbor, MI; detection limit ~12 pmol/mg protein). Tissue and plasma samples were hydrolysed (10 M NaOH), neutralized (1 M HCl) then centrifuged (3060 × g) to obtain supernatants used for total (combined free and esterified) F_2_-isoprostane analysis. All tissue lipid-soluble biomarkers were normalized to corresponding homogenate protein levels.

### Preparation of cDNA

Subsequent to assessment of leukocyte adhesion, mouse aortae were frozen, and transported on dry ice to the Charles Perkins Center (University of Sydney). Total RNA from kidney tissues or aorta was extracted from clarified homogenates with a GeneElute Mini Kit (Sigma Aldrich, Castle Hill, Australia). Complementary DNA was transcribed with BioScript Reverse Transcriptase (Bioline, Gladesville, Australia) using a MasterCycler system (Eppendorf, Australia). Reaction mixtures containing 2 μL total RNA, 2 μL oligo (dT), and 8 μL DEPC-treated Nanopure water were denatured (70°C, 5 min), chilled to 4°C (5 min), then treated with 0.25 μL RNase inhibitor (10U). Next, the mixture was treated with 1 μL dNTP (Bioline, Sydney, Australia), 4 μL 5x reaction buffer (supplied by the manufacturer), 0.25 μL Bioscript (Bioline, Sydney, Australia) and adjusted with water to 20 μL, final volume. Samples were heated (42°C, 60 min), then the reaction was stopped by heating (70°C, 10 min) and cDNA samples stored at −20°C prior to use in gene expression studies.

#### Reverse Transcriptase PCR

Gene-specific PCRs were performed with Biomix® (Bioline, Sydney, Australia) using the primer sequences shown in Supplementary Table 2. Cycling was initiated by denaturation (94°C, 5 min) followed by 25–33 cycles that involved denaturation (94°C, 30 s), annealing (0.5–1 min) and elongation (72°C, 1 min) then terminated by a final extension step (72°C, 10 min). Amplified cDNA mixed with 5x loading dye (Bioline, Australia) was resolved on 1% w/v agarose gels containing ethidium bromide (2 μg/mL). Products were visualized and imaged under short-wavelength UV light in a G-Box (BioRad, Australia) and converted to TIF files using standard imaging software. Semi-quantitative densitometry was carried out using Image J software (*v*1.42) made available in the public domain from the NIH, USA (39).

### Assessment of 3-chloro-Tyrosine/Tyrosine Ratios in Renal Tissues

Renal tissue homogenates were delipidated, then proteins precipitated with 3% w/v sodium deoxycholate and 50% w/v trichloroacetic acid and collected by centrifugation (6000 × g, 5 min). Pellets were washed with acetone, centrifuged (6000 × g, 34 min), dried under a stream of nitrogen gas, resuspended in 6 M methanesulfonic acid containing 0.2% tryptamine and hydrolyzed under vacuum (110°C, 12 h) (40).

Detection and quantification of 3-chlorotyrosine (3-Cl-Tyr) was performed by quantitative mass spectrometry. Briefly, 3-chloro-[^13^C9,^15^N] tyrosine and [^15^N]-tyrosine (final concentration 1.5 μM) were added to homogenate samples as internal standards before hydrolysis using established protocols (41, 42). Hydrolysates were purified using solid-phase extraction columns (Supelco, Sydney, Australia) activated with 100% methanol before preconditioning (2 × 2 mL) with 0.1% v/v TFA/H_2_O. Hydrolysates were loaded onto extraction columns with 2 mL 0.1% v/v TFA/H_2_O, eluted with 80% v/v methanol/H_2_O, dried under vacuum at 60°C, and re-dissolved in 100 μL 0.1% v/v formic acid. Unmodified tyrosine and 3-Cl-Tyr were detected using an LC-MS (triple quadropole) system (Agilent, Sydney, Australia) and quantified using calibration curves constructed with authentic Tyr and 3-Cl-Tyr and their corresponding isotopically-labeled isoforms (0–500 pmol) to yield the 3-Cl-Tyr/Tyr ratio.

### Cytokine Array

Quantification of cytokines: IL-1α, IL-1β, IL-2, IL-3, IL-4, IL-5, IL-6, IL-10, IL-12p70, IL-17, MCP-1, IFNγ, TNFα, MIP-1α, GMCSF, RANTES, in clarified kidney homogenates was performed with a Q-Plex Mouse IR Cytokine-Array kit (16-plex; Quansys Biosciences, Logan, UT). Total protein was normalized in all samples prior to dilution (1:1 v/v) with assay buffer containing the DyLight®IR Dye provided. Mixtures were then processed as per manufacturer's instructions and analyzed using an Odyssey-CLx®, IR light reader. Cytokine concentrations were determined by comparisons with standard curves generated under identical conditions; where a response was above the maximum detection limit (of the highest standard concentration) the change was not reported as it was unclear whether signal saturation had occurred.

### Western Blotting

Clarified kidney homogenates normalized for protein content, were diluted (50*x*) in water mixed with an equal volume of Laemmli sample buffer (2*x*, Bio-Rad, Australia) and heated (95°C, 5 min). Samples were loaded into a 12% w/v TGX Stain-Free FastCast polyacrylamide gel (Bio-Rad, Australia), and proteins were separated using a Mini-Protean Tetra electrophoresis system (Bio-Rad, Australia), and imaged with a ChemiDoc MP System (Bio-Rad, Sydney, Australia) for densitometric analyses of total protein. Proteins were then transferred to a PVDF membrane (Merck-Millipore, Kilsyth, Australia) using a TransBlot Turbo transfer system (Bio-Rad) then blocked overnight. Membranes were probed with a polyclonal anti-NFκB-P65 (phospho-Ser276) antibody and reactivity detected with goat anti-rabbit IgG HRP (Sigma-Aldrich, Castle Hill, Australia; cat#A6154) (Supplementary Table 1). Target proteins were visualized using Luminata Forte (Merck-Millipore, Kilsyth, Australia) in a ChemiDoc XRS+ System, analyzed, and quantified with Image Lab software (*v5.1*) and finally protein-normalized values were expressed as a fold-change relative to the corresponding control-group (arbitrarily assigned unitary value).

Assignment of NFκB P65 phosphorylation (P-P65) was validated by enzymatically dephosphorylating renal homogenate samples in parallel. Thus, selected samples were treated with either 12 μL H_2_O (control), 6 μL calf intestinal alkaline phosphatase (CIP) solution diluted with 6 μL H_2_O, or 12 μL undiluted CIP solution. Final concentrations of MgCl_2_ and dithiothreitol were adjusted to 10 and 1 mM, respectively, then samples incubated (2 h, 35°C). Proteins were resolved on SDS-PAGE and probed with an antibody raised against NFκB P-P65 (Supplementary Table 1) using Western blotting as described above.

### Statistical Analysis

Statistical and best-fit analyses were performed using GraphPad Prism (v 7); data was evaluated using Mann–Whitney–Wilcoxon test to account for a non-parametric distribution of data sets. Statistical significance was accepted at the 95% confidence level (*P* < 0.05) indicating that the mean value of the ranked data differed between the treatment groups that were analyzed.

## Results

### Pro-inflammatory and Pro-thrombotic Activities of Recombinant SAA

**Figure 1:**
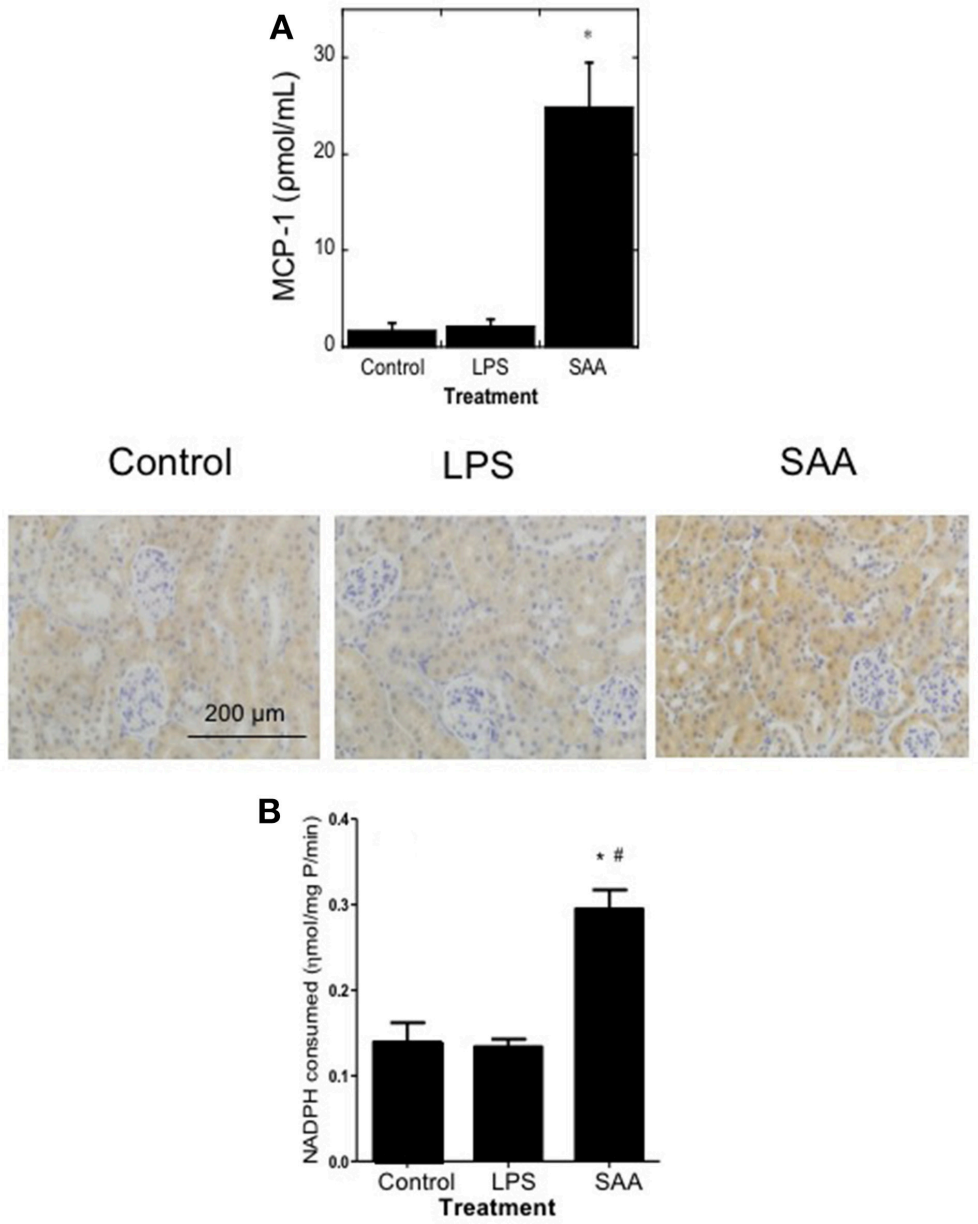
Plasma MCP-1 and renal antioxidant Gpx-1 protein and activity are elevated in mice treated with SAA. ApoE^−/−^ mice were administered SAA, LPS or sterile PBS (vehicle control) by *i.p*. injection every 3 days over 2 weeks (as described in Study 1). Plasma was isolated 4 weeks after commencement of SAA treatment and levels of **(A)** MCP-1 by commercial ELISA. Data represent mean ± SD, *n* = 8 (control and LPS groups), or 10 (SAA group) animals. ^*^Different to vehicle-control and LPS-treated mice in the absence of SAA; *P* < 0.05. Renal sections were then stained with anti-GPx1 antibody in batches so that all reagents and handling were identical, as described in Methods. Immuno-reactivity of representative kidney sections from control, LPS, and SAA-treated mice are shown. Figures represent at least two independent samples from each treatment group; magnification, x 200; scale bar, 200 μm. Renal homogenates were tested for total GPx activity **(B)**. Data expressed as means ± *SD* for control (*n* = 5), LPS (*n* = 8), or SAA (*n* = 4) treatments. *Different to the vehicle-control group; *P* < 0.05; ^#^Different to the LPS group; *P* < 0.05.

Levels of pro-inflammatory, pro-thrombotic, and antioxidant genes in aortic and renal tissues from ApoE^−/−^ mice that were administered either vehicle, a dose of LPS similar to contaminating levels in recombinant SAA, or SAA every 3 days for 2 weeks then harvested after a further 2 weeks are shown in Table 1. Consistent with the reported pro-inflammatory and pro-thrombotic activities of SAA (10, 15, 30), thoracic aortae contained elevated NFκB and tissue factor (TF) mRNA compared to controls whereas these genes were not increased in samples from mice given LPS. Activation of an inflammatory response was confirmed by the significantly increased (~12-fold) in plasma MCP-1 from SAA-treated mice (*p* < 0.05 compared to control or LPS; Figure 1A).

**Table 1:** Gene expression in aortic and renal tissues.

**Treatment Group**	**Control (*n* = 7)**	**LPS (*n* = 8)**	**SAA (*n* = 6)**
**AORTA**			
NF-κB	1.0 (0.0)	1.1 (0.1)	1.6 (0.1)^*^^#^
TF	1.0 (0.1)	1.2 (0.1)	2.3 (0.4)^*^^#^
GPx-1	1.0 (0.1)	1.2 (0.2)	1.3 (0.6)
CAT	1.0 (0.2)	1.1 (0.2)	2.4 (0.6)^*^^#^
**KIDNEY**			
GPx-1	1.0 (0.1)	1.0 (0.3)	2.9 (1.2)^*^^#^
CAT	1.0 (0.1)	1.3 (0.2)	3.4 (0.6)^*^^#^

*Mice received vehicle, LPS or SAA every 3 days over 14 days. After a further 2 weeks, mice were euthanized and total mRNA isolated from aortae and kidneys. Corresponding cDNA products were probed for gene expression by RT-PCR. mRNA expression was normalized to β-actin house-keeping gene and arbitrarily assigned a value of 1 and other data expressed relative to this level; data represent means ± (SD)*.

* *Different to control; P < 0.05*.

# *Different to the corresponding LPS group; P < 0.05. 4 samples of kidney homogenate from the SAA-treatment group failed to yield suitable cDNA product for PCR analyses*.

The endogenous enzymic antioxidant catalase (CAT), but not glutathione peroxidase-1 (GPx-1) mRNA was significantly increased in aortae although both were elevated in renal tissue 4 weeks after commencement of SAA treatment (Table 1). In contrast, LPS administration did not significantly alter expression of aortic or kidney mRNA levels of any genes tested compared to vehicle-treated controls (Table 1). Immunohistochemical analysis of GPx-1 reactivity in kidneys from Apo E^−/−^ mice 4 weeks after commencement of SAA treatment indicated low-level constitutive expression in renal tubular epithelium with similar expression patterns in control and LPS treated mice (Figure 1). In contrast, GPx-1^+^ immune-staining was relatively intense in renal tubular epithelia from ApoE^−/−^ mice administered SAA (Figure 1), which corresponded to the significantly increased total GPx activity determined in matched renal homogenates (Figure 1B). Thus, in addition to promoting a pro-inflammatory response in renal tissues, SAA stimulated an up-regulation of CAT (gene), and GPx (gene, protein, and activity) in the kidney, both enzymes responsible for metabolizing hydrogen peroxide (43).

Oxidative damage in ApoE^−/−^ mice that received SAA was further examined by comparing native and oxidized lipid levels in plasma (Table 2) and in renal tissue (Table 3), with those from vehicle or LPS-treated controls sampled 4- or 18-weeks after treatment commenced. Tissue lipid and vitamin E content was similar across all groups at both time points (corresponding plasma levels of free cholesterol; FC, and vitamin E; TOH and polyunsaturated lipids C20:4 and C18:2 were similar between treatment groups; *P* > 0.05; suggesting that none of the treatments altered plasma levels of lipids or the ratio of lipid-to- lipid soluble antioxidant; TOH). However, SAA induced significant lipid (per)oxidation; thus CE-O(O)H) increased in plasma [0.4 ± 0.3 (SAA) vs. 0.01 ± 0.01 (control) nM; mean ± SD; *P* < 0.05] and renal tissue [18.3 ± 4.7 (SAA) vs. 4.0 ± 2.3 (control) pmol/mg protein; mean ± SD; *P* < 0.05] 4 weeks after commencement of treatment and similarly increased in plasma [0.4 ± 0.3 (SAA) vs. 0.01 ± 0.01 (control) nM; mean ± SD; *P* < 0.05] and renal tissue [24.4 ± 17.7 (SAA) vs. 1.4 ± 2.0 (control) pmol/mg protein; mean ± SD; *P* < 0.05] 18 weeks after commencement of SAA-treatment, relative to control or LPS groups. Notably, F_2_-isoprostanes levels (an independent biomarker of free radical-mediated arachidonic acid oxidation) were also elevated significantly in samples from mice given SAA vs. vehicle and LPS controls, and generally increased in parallel with CE-O(O)H levels in plasma and tissue measured at 4 or 18 weeks after commencement of treatment (Tables 2, 3). This outcome indicates that SAA initiates and sustains oxidative stress in both the vasculature and the kidney.

**Table 2:** Plasma concentrations of native and oxidized lipids and vitamin E^a^.

	**Control^b^**	**LPS**	**SAA**	**Control**	**LPS**	**SAA**
	***n*** **= 8**	***n*** **= 8**	***n*** **= 10**	***n*** **= 8**	***n*** **= 8**	***n*** **= 10**
	**4 weeks after starting SAA-treatment**			**18 weeks after starting SAA treatment**		
[FC] mM	3.5 (1.5)	3.0 (0.5)	3.0 (0.9)	3.5 (0.8)	3.2 (0.4)	3.3 (0.7)
[TOH] μM	5.0 (2.9)	4.2 (2.5)	4.7 (2.8)	4.6 (1.9)	4.5 (1.6)	4.8 (1.6)
[CeO(O)H] nM	0.01 (0.01)	0.02 (0.02)	0.4 (0.3)^*^^#^	0.01 (0.02)	0.03 (0.02)	0.6 (0.1)^*^^#^
[C18:2] mM	0.4 (0.2)	0.4 (0.1)	0.6 (0.2)	0.5 (0.2)	0.6 (0.1)	0.5 (0.2)
[C20:4] mM	1.4 (0.7)	1.3 (0.3)	1.6 (0.6)	1.2 (0.4)	1.3 (0.3)	1.6 (0.7)
[F_2_-isoprostanes] pg/mL	35.1 (5.7)	38.0 (6.9)	81.9 (7.8)^*^^#^	41.8 (19.2)	36.1 (10.1)	52.8 (9.1)

a *Mice were given vehicle, LPS or SAA every 3 days over 14 days by i.p. injection (see Materials and Methods). At 4 or 18 weeks after SAA treatment, blood was isolated, and plasma prepared, and the parameters listed above were measured by liquid chromatography or an ELISA kit (F_2_-isoprostanes). Data represent means ± (SD); n–as indicated. Unesterified cholesterol, FC; α-tocopherol (biologically active vitamin E), α-TOH; cholesteryl linoleate, C18:2; cholesteryl arachidonate, C20:4; combined cholesteryl esters (C18:2 and C20:4), CE; CE-derived lipid hydroperoxides and hydroxides, CeO(O)H*.

b *Units of measurement are as indicated*.

* *Different to control; P < 0.05*.

# *Different to the corresponding LPS group; P < 0.05*.

**Table 3:** Kidney tissue concentrations of native and oxidized lipids and vitamin E^a^.

	**Control^b^**	**LPS**	**SAA**	**Control**	**LPS**	**SAA**
	***n*** **= 8**	***n*** **= 8**	***n*** **= 10**	***n*** **= 8**	***n*** **= 8**	***n*** **= 10**
	**4 weeks after starting SAA-treatment**			**18 weeks after starting SAA-treatment**		
[FC] nmol/mg P	1.7 (0.3)	1.6 (0.4)	1.7 (0.3)	1.9 (0.5)	1.9 (0.5)	1.8 (0.6)
[TOH] pmol/mg P	127.8 (30.9)	132.9 (31.7)	125.2 (42.9)	142.2 (60.2)	138.2 (46.1)	154.6 (55.8)
[CeO(O)H] pmol/mg P	4.0 (2.3)	1.9 (0.9)	18.3 (4.7)^*^^#^	1.4 (2.0)	2.7 (1.4)	24.4 (17.7)^*^
[C18:2] nmol/mg P	0.3 (0.02)	0.3 (0.02)	0.3 (0.3)	0.4 (0.2)	0.4 (0.2)	0.5 (0.2)
[C20:4] nmol/mg P	0.9 (0.4)	1.1 (0.6)	1.0 (0.5)	2.1 (2.2)	1.2 (0.4)	1.6 (0.7)
[F_2_-isoprostanes] pg/mg P	21.2 (4.7)	28.5 (9.6)	66.9 (7.8)^*^, ^#^	35.3 (7.1)	40.2 (7.1)	51.8 (6.1)^*^^#^

a *Mice received normal chow and were administered vehicle, LPS or SAA every 3 days over 14 days by i.p. injection (for details see Materials and Methods). At 4 or 18 weeks after SAA treatment, kidneys were harvested, and the following parameters measured in prepared renal tissue by liquid chromatography or an ELISA kit (F_2_-isoprostanes). Unesterified cholesterol, FC; α-tocopherol (biologically active vitamin E), α-TOH; cholesteryl linoleate, C18:2; cholesteryl arachidonate, C20:4; combined cholesteryl esters (C18:2 and C20:4), CE; CE-derived lipid hydroperoxides and hydroxides, CeO(O)H*.

b *Units of measurement are as indicated. Data represent Means ± (SD); n–as indicated*.

* *Different to the control; P < 0.05*.

# *Different to the corresponding LPS group; P < 0.05*.

### SAA-Stimulates Pro-atherogenic Changes to the Vasculature

**Figure 2:**
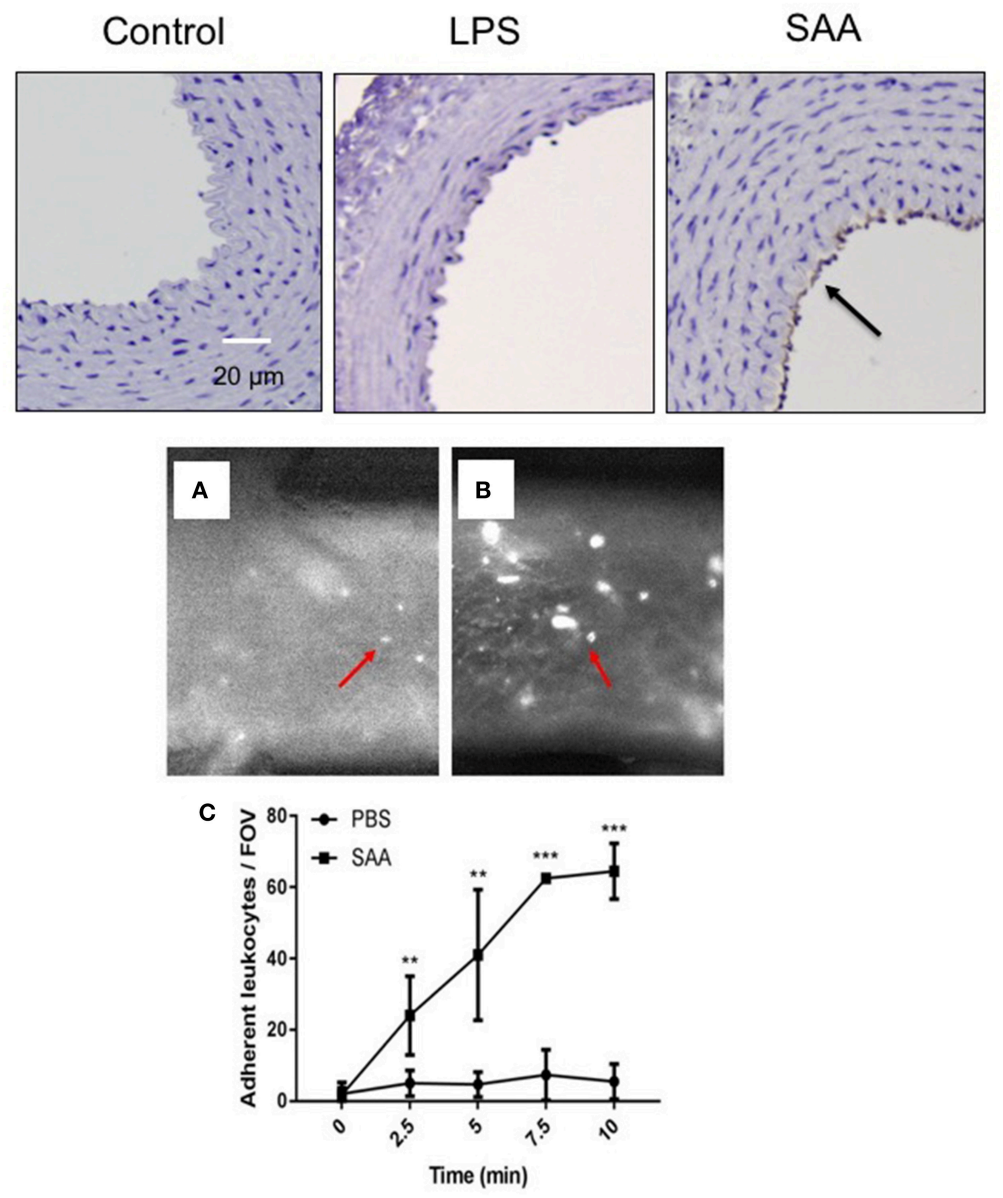
SAA enhances vascular cell adhesion molecule (VCAM) expression in ApoE^−/−^ mice leading to increased adhesion of circulating leukocytes. Upper panels: ApoE^−/−^ mice were injected *i.p*. with SAA, LPS or sterile PBS vehicle and sacrificed 2 weeks after treatment was finalized (as described under Study 1; experimental design). This sections of thoracic aorta from vehicle (control)-, LPS-, and SAA-treated mice were stained with anti-VCAM-1, counterstained with hematoxylin then imaged by light microscopy (arrow indicates immune-reactive VCAM-1). Data is representative of aortae from *n* = 6 mice in control and SAA groups and *n* = 4 mice in the LPS-treatment group. Scale bar = 50 μm. Lower panels **(A,B)**: Aortae were isolated from ApoE^−/−^ mice treated with sterile PBS vehicle or SAA (as described under Study 2; experimental design). Aortae were carefully cleaned of fat and placed in an *ex vivo* dynamic flow adhesion system to assess the adherence of fluorescently labeled leukocytes using a live stage fluorescent microscope as described in Methods. **(C)** The total number of cells adherent to the vessel wall quantified time-dependently. Data represent mean ± *SD, n* = 4 mice per group. ^**^Different to the vehicle control; *P* < 0.05; ^***^Different to the vehicle control; *P* < 0.05.

VCAM-1 expression on the vascular endothelium indicates activation of a pro-adhesive state that promotes the translocation of monocytes to the subendothelial space, an early marker for atherogenesis. In keeping with data shown in Figure 1, this was not detected on aortae from ApoE^−/−^ mice exposed to LPS or vehicle (Figure 2) for the same period. In contrast, VCAM-1 expression was evident in the endothelium of aortae 4 weeks after commencement of SAA-treatment, indicating early pro-inflammatory and pro-atherogenic changes in the arterial wall (Figure 2). Next, the aortae used for *ex vivo* flow studies were assessed for VCAM-1 gene expression. Consistent with the increased VCAM-1^+^ immune reactivity detected 4 weeks after SAA administration, VCAM-1 mRNA also markedly increased (~3.7-fold increase; *P* < 0.05, *n* = 4 mice), compared to the vehicle control (Supplementary Figure 1). Consistent with this pro-atherogenic action, SAA significantly increased leukocyte adhesion in aortic vessels isolated from ApoE^−/−^ mice administered SAA whereas the corresponding control mice (sterile PBS as a vehicle control) did not (compare Figure 2A,B). Overall, SAA enhanced leukocyte adherence to the vessel wall in a time-dependent manner relative to the control (Figure 2C).

### SAA Stimulates Changes in Vascular Endothelial Cell and Renal Function

**Figure 3:**
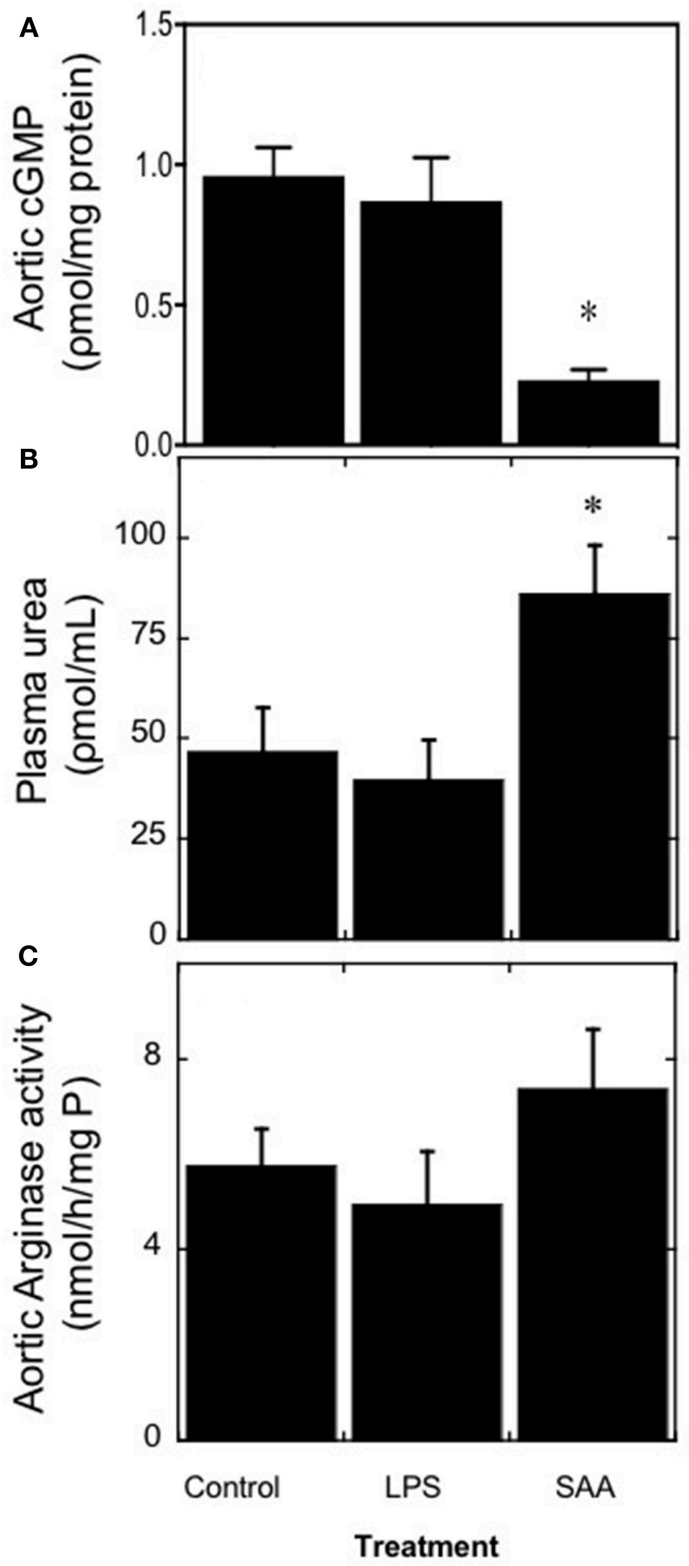
SAA increases circulating urea levels in ApoE^−/−^ mice. Male ApoE^−/−^ mice were administered SAA, LPS or sterile PBS (vehicle control) by *i.p*. injection over 2 weeks and sacrificed a further 2 weeks after cessation of treatment (as described in Study 1). Aortae were excised and homogenized and then the homogenate samples tested for the levels of **(A)** cGMP as a surrogate marker for vaso-dilating NO. Plasma levels of **(B)** urea were quantified and their corresponding isolated aortae **(C)** assessed for total arginase activity as described in Methods. Data represent mean ± *SD, n* = 8 (control and LPS groups), or 10 (SAA group) animals. *Different to vehicle-control and LPS-treated mice in the absence of SAA; *P* < 0.05.

The secondary effector molecular cGMP was measured as a surrogate for the NO-soluble guanylyl cyclase-cGMP vasomotor axis (Figure 3A). Overall, cGMP was significantly diminished in aortic samples 4 weeks after commencement of SAA treatment whereas, aortic homogenates from the vehicle- and LPS-treated controls contained similar levels. By contrast, SAA-treatment significantly increased urea in plasma samples from ApoE^−/−^ mice 4 weeks after commencement of SAA treatment relative to the controls (Figure 3B). Our earlier study indicated that SAA enhances arginase (Arg)-1/2 expression in cultured endothelial cells and may potentially impact on NO bioavailability, promoting endothelial dysfunction; Arg-1/2 metabolizes *L*-arginine to yield urea and *L*-ornithine (16). However, although aortic Arg-1/2 activity trended to increase in SAA-treated mice this was not significantly different greater than levels in aortae from vehicle or LPS-treated controls (Figure 3C) suggesting an alternate mechanism for the SAA-stimulated increase in plasma urea.

**Figure 4:**
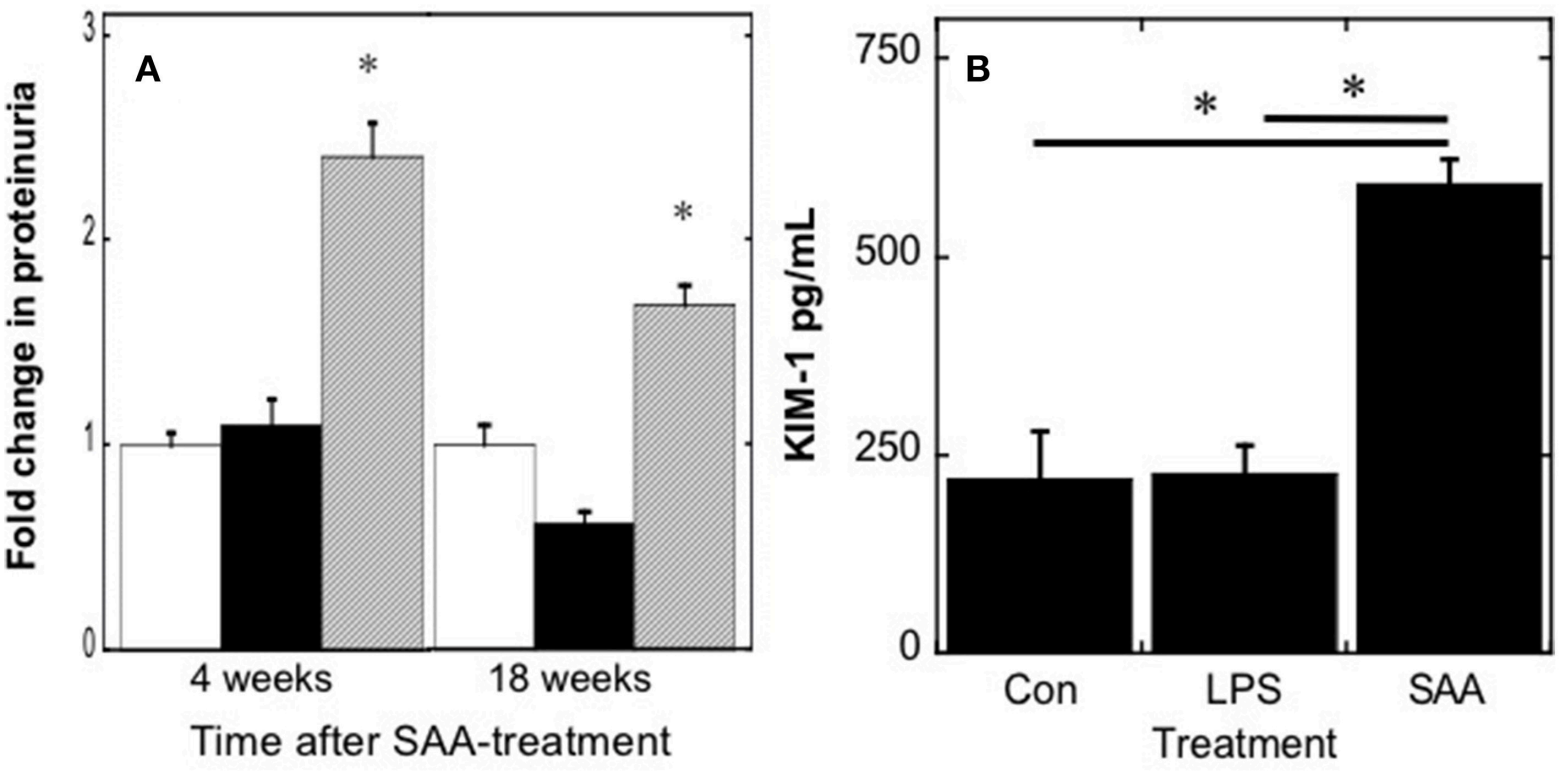
Urinary protein and kidney injury molecule-1 increase in mice after SAA administration. ApoE^−/−^ mice were treated with SAA, LPS or sterile PBS by *i.p*. injection over 2 weeks (as described in Study 1). Total protein **(A)** was quantified in urine samples from mice 4 or 18 weeks after commencement of SAA-treatment; open, black and gray bars represent control, LPS and SAA-treated groups. To assess acute renal damage, **(B)** urinary KIM-1 concentrations were determined in samples obtained 4 weeks after commencement of SAA-treatment using a commercial ELISA kit. Data represent mean ± *SD*; *n* = 8 (control and LPS) or 10 (SAA) mice. *Different to control and LPS-treated mice in the absence of SAA; *P* < 0.05.

Increased blood urea nitrogen products may result from increased vascular Arg-1/2 activity, increased protein catabolism, or possibly decreased glomerular filtration. As aortic Arg-1/2 activity was similar between treatment groups (Figure 3C), we investigated the impact of SAA on some indices of renal damage (Figure 4). SAA significantly increased urinary protein (Figure 4A) and elevated levels of urinary KIM-1 (Figure 4B) 4 weeks after commencement of treatment. Urinary protein remained elevated in ApoE^−/−^ mice [*P* < 0.05; *n* = 8 (control and LPS groups) *n* = 10 (SAA group)] 18 weeks after completing SAA treatment, although somewhat less than total protein levels found in urine from mice culled 4 weeks after commencement of SAA treatment (see Figure 4A). Taken together, these data suggest that SAA-mediated damage to the kidney impacts on renal function and may contribute to the increase in blood urea levels.

### SAA Stimulates Pathological Changes in Renal Tissues

**Figure 5:**
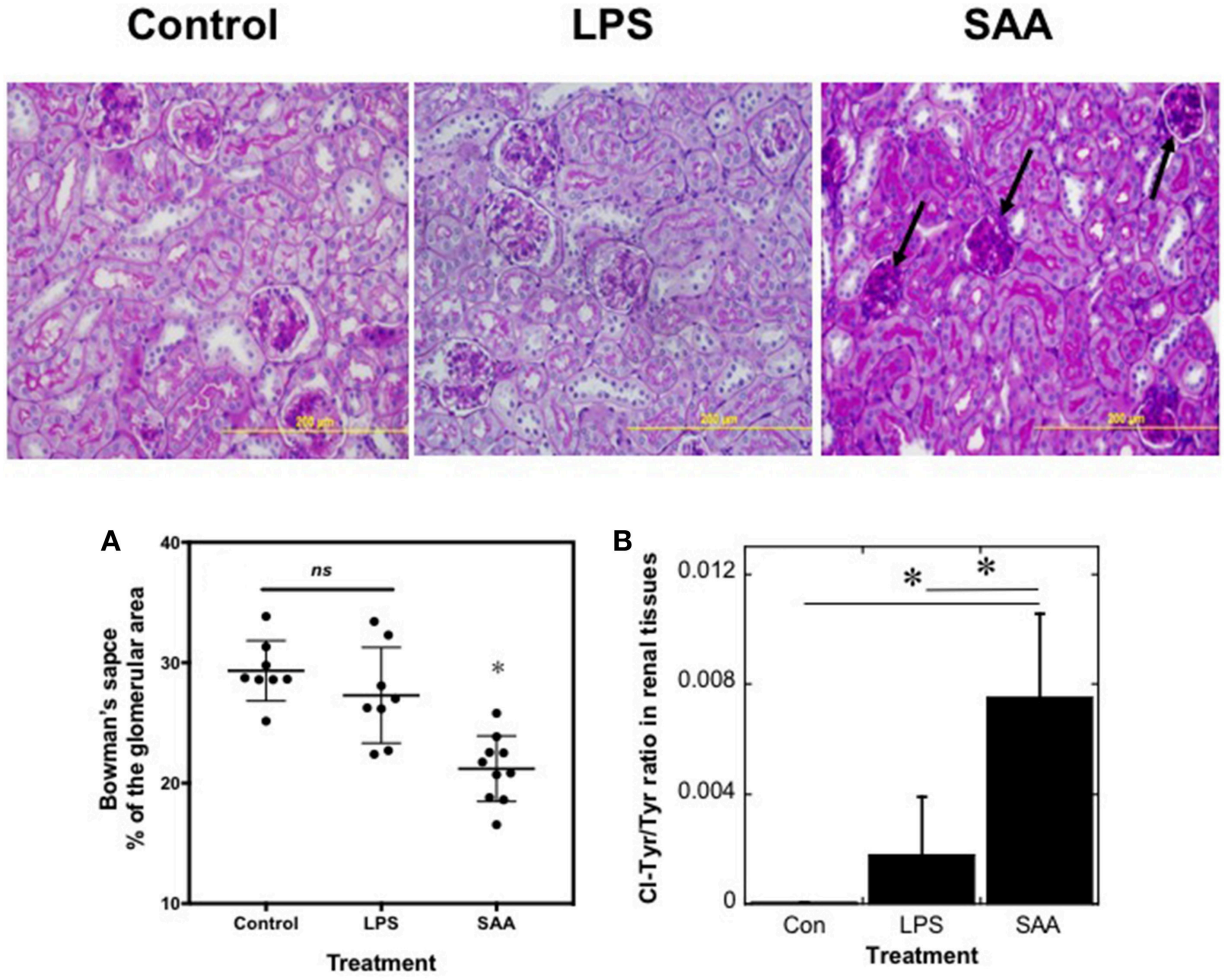
SAA promotes kidney inflammation in ApoE^−/−^ mice. ApoE^−/−^ mice were administered SAA, LPS or sterile PBS for 2 weeks (as described in Study 1). After a further 2 weeks mice were sacrificed and kidney sections from control, LPS- and SAA-treated mice were stained with PAS. Data represent at least 3 different fields within each kidney section obtained from *n* = 8 (control or LPS) or 10 (SAA) animals. Arrows highlight condensed glomeruli in renal samples from SAA-treated mice. Magnification *x*200, scale bar = 200 μm. The change in Bowman's space **(A)** expressed as a percentage of the corresponding total glomerular area was calculated as defined in the Methods. Each point represents mean data from glomeruli present in a field of view (at least 3 fields of view; FOV) obtained at 200x magnification as assessed with Image-pro Plus (V6). Data represent mean ± *SD*; *n* = 8 (Control) or 8 (LPS-) and 10 SAA-treated mice. ^*^Different to vehicle- or LPS-treated mice in the absence of SAA; *P* < 0.05; *ns*, not significant. Renal homogenates were assayed simultaneously for 3-chlorotyrosine and total tyrosine content **(B)**. Quantitative mass data represent mean ± *SD*; *n* = 5 (Control) or 4 (LPS- and SAA-treated) mice. ^*^Different to vehicle- or LPS-treated mice in the absence of SAA; *P* < 0.05.

To assess changes in renal architecture, PAS-stained kidney sections were examined (Figure 5, Upper). Kidneys harvested from SAA-treated animals at 4 weeks after commencement of SAA treatment showed evidence of diffuse glomerular injury, with hypercellularity, condensed glomeruli, and thickened capillary walls within the glomerular tufts (Figure 5, black arrows), changes not seen in kidneys from mice exposed to low-level LPS or vehicle Consistent with SAA stimulating an increased glomerular cellularity, cortical glomeruli showed a significant decrease in the fraction of Bowman's space, relative to total area occupied by the corresponding glomerulus (decreased ~7%; *P* < 0.0001) when compared to the control and LPS-treated groups, which were not different (Figure 5A). In contrast, renal architecture appeared normal in kidneys from all treatment groups harvested at 18 weeks (not shown) and there was no evidence of renal AA amyloidosis in kidneys from SAA-treated mice in the older groups of mice (Supplementary Figure 2).

The glomerular changes may be the consequence of oxidative damage to the kidneys. To examine this possibility, the proportion of 3-chlorotyrosine (3-Cl-Tyr), relative to total tyrosine (i.e., the ratio 3-Cl-Tyr/Total Tyr), a biomarker of HOCl production that correlates with MPO activity (44), was assessed in kidney samples from all treatment groups using liquid chromatography coupled with mass spectrometry (41, 42) (Figure 5B). The ratio of 3-Cl-Tyr/Total Tyr was elevated significantly in kidneys from SAA-treated mice and represented ~0.8% of the total tyrosine residues in proteins within these samples. In marked contrast, 3-Cl-Tyr was below the level of detection in controls and the 3-Cl-Tyr/Total Tyr ratio increased only marginally in kidneys from LPS-treated mice.

**Figure 6:**
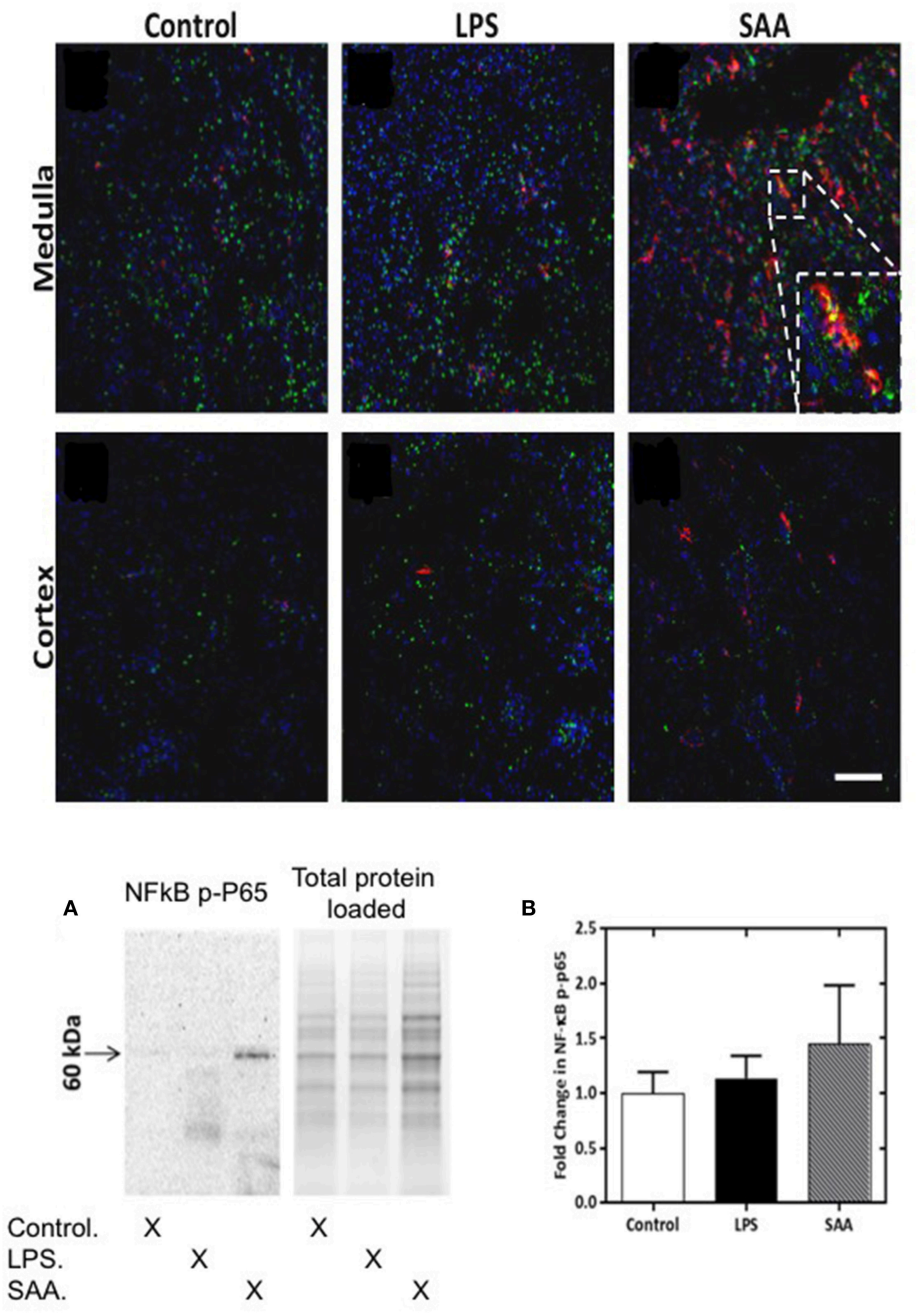
NFκB phospho-p65 is increased in SAA-treated ApoE^−/−^ mice kidney. ApoE^−/−^ mice were administered SAA, LPS or sterile PBS and kidneys harvested 3 days later. Tissue sections were incubated with anti-mouse NFκB *P*-P65 Ser267 (FITC) and anti-mouse F4/80 (Alexafluor 594). Representative views (*x*200 magnification) are shown for renal medulla (Upper and cortex (Lower) from control, LPS- and SAA-treated mice. Where NFκB *P*-P65^+^ immunoreactivity (Ser267) labeling (green) co-registered with F4/80^+^ immunoreactivity labeling (red) cells appear yellow. Figure inset shows magnified region highlighting NF-κB *P*-P65^+^ and F4/80^+^ immunoreactivity and co-registration of these antigens in the renal medulla. Scale bar = 100 μm. NK-κB *P*-P65 levels in SAA-treated ApoE^−/−^ mouse kidney tissue homogenates were examined by Western blot **(A)** and normalized to total (in-gel) protein as shown in **(B)**. Data are means ± *SD*; *n* = 2 experiments performed in duplicate.

Neutrophils and macrophages produce MPO and HOCl (45, 46). To assess whether these cells were present in renal infiltrates, we conducted an immunohistochemical assessment of matched kidney tissues using neutrophil- and macrophage-specific antibodies. We were unable to detect Ly6G^+^ neutrophils in kidneys from ApoE^−/−^ mice harvested at 3–5 days, or at 28 days post SAA-treatment (Supplementary Figure 3). Although F4/80^+^ macrophages were numerous in the kidney (particularly the medulla) (Figures 6A–C), almost none exhibited MPO^+^ co-staining (*c.f*., Supplementary Figure 4A–F shown at 200x magnification).

Macrophages can be stimulated and recruited directly by SAA (47) and also by MCP-1 [via SAA activating TLR2/4SR-BI receptor pathways (48)], which we show here increased in the circulation following SAA administration (Figure 1A). To assess macrophage activation, we determined the extent of NFκB activation by assessing phosphorylated P65 NFκB subunit (NFκB *P*-P65) as a surrogate marker. For all mice, NFκB *P*-P65^+^ immune-positive staining was detected as a punctate distribution within the renal medulla (Figure 6). F4/80^+^ immune-positive cells were more frequent in the medulla of kidneys from SAA-treated ApoE^−/−^ mice 5 days post treatment compared to corresponding tissue from control and LPS treated mice. Some NFκB *P*-P65^+^ immune-reactivity coincided with the location of F4/80^+^ immune-positive cells in the renal medulla, as indicated by the yellow overlay (see inset to Figure 6); this co-incident staining of NFκB *P*-P65^+^ and F4/80^+^ was more frequent in the medulla of kidneys from SAA-treated ApoE^−/−^ mice than in the corresponding tissue from control and LPS treated mice (Figures 6A–C). The extent of NFκB *P*-P65^+^ and F4/80^+^ co-incident staining was markedly lower in the cortex than the corresponding renal medulla from the same kidneys (*c.f*. Figure 6, lower panels). Although F4/80^+^ immune-labeling was somewhat higher in samples from SAA-treated mice than vehicle and LPS controls, no co-staining of NFκB *P*-P65^+^ and F4/80^+^ was evident within the cortical region. Western blotting for NFκB *P*-P65 suggested increased NFκB activation (Figure 6A) although differences estimated by densitometric analysis were not statistically significant (Figure 6B; *P*-value ~0.08). Subsequent dephosphorylation of NFκB *P*-P65 with calf intestinal phosphatase (Supplementary Figure 5) confirmed the band assignment for NFκB *P*-P65, and hence activation of NFκB assessed by Western blot.

### SAA Stimulates Production of Pro-inflammatory Cytokines and Chemokines and Tissue Factor in Renal Tissues

**Figure 7:**
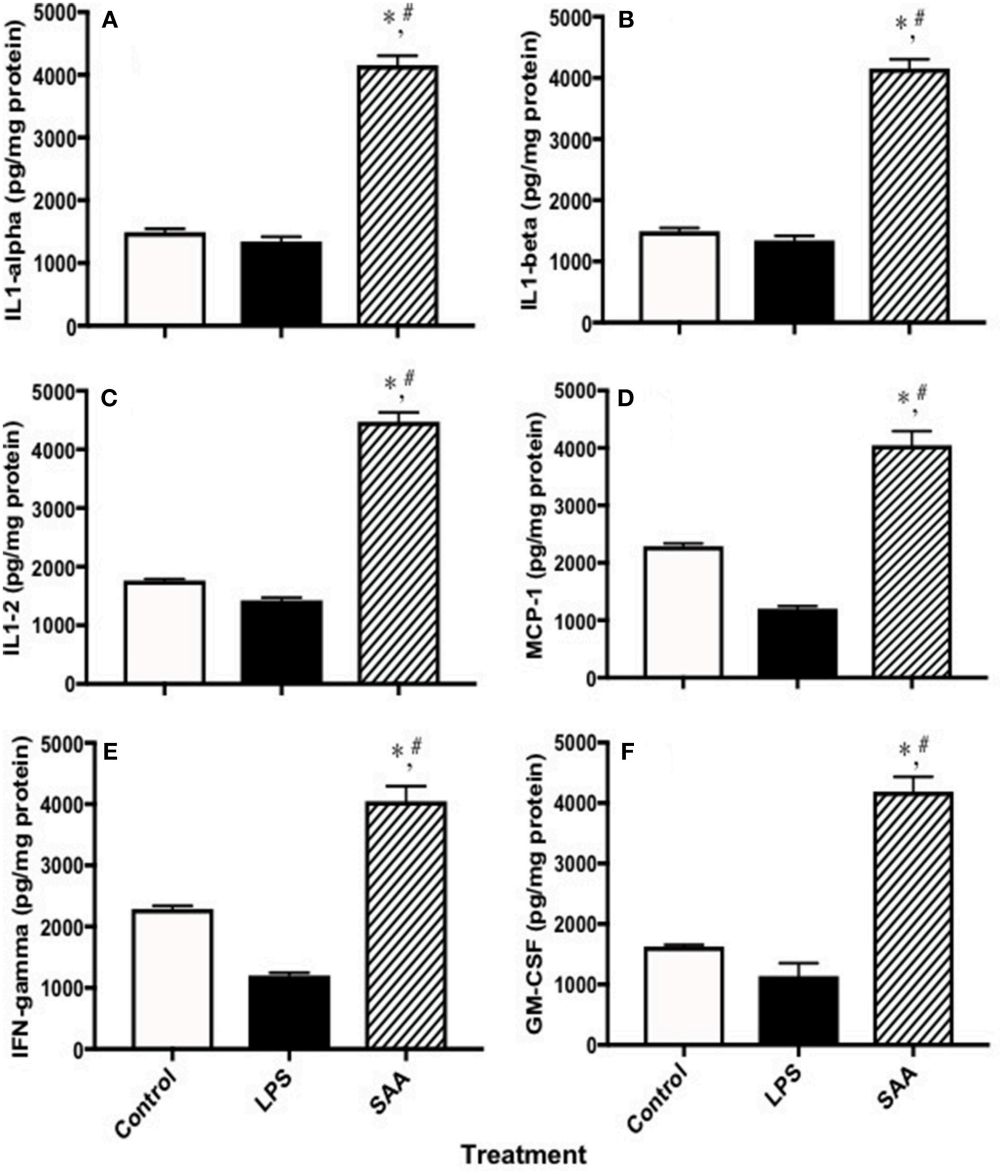
Cytokines and chemokines in kidneys from control, LPS and SAA-treated mice. ApoE^−/−^ mice were administered SAA, LPS or sterile PBS for 2 weeks (as described in Study 1). After a further 2 weeks mice were sacrificed and levels of **(A)** IL-1α, **(B)** IL-1β, **(C)** IL-2, **(D)** MCP-1, **(E)** IFN-γ, and **(F)** GM-CSF were determined in kidney homogenates with a commercial Multiplex kit. Data expressed as mean ± *SD*: control (*n* = 7), LPS (*n* = 8) and SAA (*n* = 6). *Different to the control; *P* < 0.05. ^#^Different to the LPS group; *P* < 0.05. Note, renal levels of IL-3, IL-4, IL-5, IL-6, IL-10, IL-12p70, IL-17, TNFα, MIP-1α, and RANTES that were determined on the same ELISA plate were all markedly elevated above those detected in vehicle- and LPS-treated (control) mice albeit this was above the maximum detection limit and therefore, not quantified.

To examine pro-inflammatory cytokines in kidneys from SAA-treated mice, clarified homogenates were analyzed using a multiplex system. Levels of interleukins −1α, −1β, and −2 and MCP-1, IFN-γ and GM-CSF in kidneys from mice 4 weeks after SAA treatment commenced were significantly elevated compared to corresponding control and LPS-treated samples (Figure 7), confirming a pro-inflammatory response within renal tissue. Of note, multi-plex ELISA assessment of MCP-1 levels in renal tissues from SAA-treated mice increased significantly, an outcome that was internally consistent with increased circulating MCP-1 detected in plasma from the same group of SAA-treated mice (Figure 1A). Other cytokines were also elevated in mice treated with SAA however, protein normalized values were above the maximal linear cut off range for the standards supplied (data not shown).

**Figure 8:**
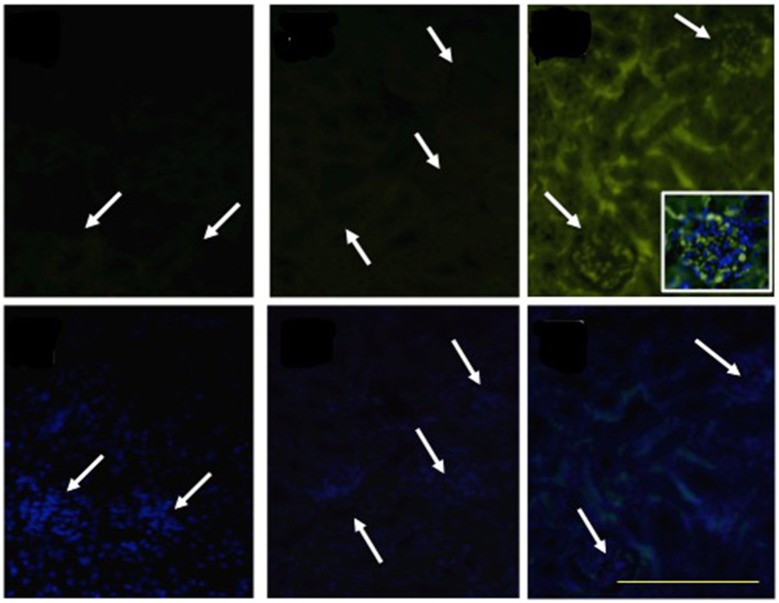
SAA promotes TF accumulation in renal tissues. ApoE^−/−^ mice were administered SAA, LPS or sterile PBS for 2 weeks (as described in Study 1). Tissue sections obtained from vehicle- (control), LPS- and SAA–treated mice were co-stained with an FITC-conjugated anti-TF antibody (Upper) or with DAPI alone (corresponding Lower) to highlight the nuclear envelope then imaged with an Olympus fluorescence microscope. No TF^+^ immune-reactivity was detected in kidney tissue from control or LPS-treated mice. Arrows indicate clusters of DAPI-labeled nuclei localized to glomeruli that are detected in lower panels and reflected as a corresponding arrow in the corresponding panel above to indicate glomeruli in the same renal section. Data are representative of at least three different fields of view (×200 magnification) from each kidney section taken from *n* = 8 (control or LPS) or 10 (SAA) mice. Digital inset shows a representative overlay of DAPI and FITC fluorescence within a glomerulus (400× magnification); scale bar, 200 μm.

Consistent with our finding that SAA increased TF gene expression in the vascular endothelium (Table 1), we identified diffuse TF^+^ immunoreactivity in kidneys harvested from ApoE^−/−^ mice 4 weeks after SAA treatment commenced (Figure 8). TF^+^ immunoreactivity was localized to glomerular tufts and tubular epithelium. Overlay of DAPI-stained cells with TF^+^ immunofluorescence indicated localization of TF protein to the glomerular capillary network in kidneys from mice receiving SAA (see inset, Figure 8). In contrast, there was little TF^+^ immunoreactivity in kidney sections from control (vehicle-) or LPS-treated mice (Figure 8; white arrows in upper panels reflect regions with clusters of DAPI staining nuclei (as detected in the corresponding lower panels), interpreted to represent glomeruli present in identical sections with TF^+^ immunoreactivity.

## Discussion

Elevated circulating SAA levels are consistently observed in conditions with a low-grade inflammatory component, such as in atherosclerosis, diabetes and obesity and are associated with increased risk of clinical coronary events (10, 11). This present study extends this body of evidence to show that administered recombinant SAA increased vascular inflammation, decreased aortic cGMP levels and impacted on renal function manifesting as increases in proinflammatory cytokines and chemokines, TF protein expression within the glomerular capillary network and increased renal oxidative stress judged by the simultaneous increase in 3-Cl-Tyr, the biomarker for the potent oxidant HOCl, elevated markers of lipid (per)oxidation, and altered gene expression of antioxidant response elements in the same renal tissues. These SAA-stimulated pathological changes to the vascular endothelium and kidney highlight a general SAA-mediated increase in inflammation. Deposition of AA amyloid was not detected in kidneys examined here. The effects of SAA occurred in ApoE deficient mice in the absence of a dietary fat load or changes in plasma or renal tissue lipids, indicating that SAA-mediated endothelial dysfunction, even in the absence of a pro-atherogenic diet, is sufficient to accelerate injury to the vasculature and the kidney.

Notably, SAA up-regulated aortic gene expression (Table 1) and subsequent phosphorylative activation of NF-κB, which may facilitate production of downstream pro-inflammatory mediators and chemokines (Figure 7) that ultimately modulate leukocyte recruitment as demonstrated by *ex vivo* vascular flow studies (Figures 2A–C). Increases in vascular pro-inflammatory factors manifested early (≤5 days) and were detected up to 4 weeks after commencement of SAA treatment. Thus, SAA stimulated increases in aortic VCAM-1 expression and plasma MCP-1 levels that could facilitate T lymphocyte and monocyte adhesion and extravasation into the vessel wall, key early events implicated in atherogenesis (49, 50). Gene deletion of SAA isoforms 1/2 in LDL-receptor-deficient (SAA^−/−^/LDL^−/−^) mice inhibited vascular lesion formation in animals receiving a high fat diet (51) again implicating a direct pro-atherogenic role for SAA. Furthermore, pro-thrombotic TF expression was elevated within glomerular capillaries and this may be ascribed directly to SAA activation of endothelial cells(52). These results have important implications, suggesting that an acute or brief elevation of SAA, potentially as a result of infection in early life, may prime individuals for endothelial dysfunction that may promote pro-atherogenic factors. Subsequently, this may facilitate development of early atherosclerotic lesions and promote arterial and renal damage subsequent to SAA-mediated activation of the endothelium as demonstrated elsewhere (53). Similarly, in conditions in which SAA levels are chronically elevated, such as diabetes (54), rheumatoid arthritis (9, 25), and chronic renal dysfunction (55, 56); susceptibility to SAA-mediated endothelial damage may promote CVD and related vascular complications.

Here we show that short-term treatment with SAA caused kidney injury and renal impairment in ApoE^−/−^ mice on a normal chow diet. Thus, SAA activated the aortic endothelium and potentially the renal microvasculature, manifesting as expression of VCAM-1 in the thoracic aorta, localized inflammation in renal glomeruli and kidney tissue damage, the latter indicated by increased urinary KIM-1 and sustained proteinuria. Even mildly reduced kidney function (manifesting as micro-albuminuria) is an established risk factor for progression to renal disease and cardiovascular events (56, 57), thereby linking elevated circulating SAA levels with early stage renal dysfunction and pre-clinical atherosclerosis.

NFκB activation is pivotal for transcription of many pro-inflammatory genes that promote endothelial dysfunction and initiate fibrotic responses, thrombosis, and associated pathological changes in organs such as the kidney. Increased NFκB *P*-P65^+^ immunofluorescence indicated elevated NFκB activity in the medulla of kidneys isolated from ApoE^−/−^ mice 5 days post SAA administration, and at least within the renal medulla, was associated with F4/80-positive macrophages, suggesting that macrophage activation may contribute to NFκB phosphorylation. Moreover, TF mRNA, an NFκB-dependent product, increased with a concomitant increase in TF protein in glomerular capillary tufts and within tubular epithelium implying that SAA can stimulate procoagulant activity through an NFκB-dependent pathway, a feature of several kidney pathologies (58). Key pro-inflammatory cytokines (IL-1α, IL-1β, IL-2, IFN-γ, GM-CSF, MCP-1) were also significantly increased in kidney homogenates from SAA-treated mice. Concomitant with increased renal MCP-1 accumulation, F4/80^+^ macrophages appeared to increase in the renal medulla from ApoE^−/−^ mice receiving SAA for only 3 or 5 days, suggesting increased monocyte recruitment (and subsequent maturation to yield macrophages) in this region of the kidney. This notion is supported by the observation that glomeruli also showed evidence of cellular recruitment with the % of Bowman's space relative to the corresponding glomerular area shown to be significantly decreased in renal sections from the SAA-treatment group (Figure 5A), suggesting that SAA-mediated activation of the endothelium localized to glomerular capillaries stimulated cellular recruitment. In contrast, no neutrophil influx was detected in the corresponding renal tissues at these times. Although MPO activity is the only plausible explanation for the increased renal 3-Cl-Tyr observed 4 weeks after SAA treatment commenced, we were unable to determine its cellular source. One possible explanation for this is that proteins modified by MPO/HOCl may persist in tissues after transient MPO activity has subsided, and our sampling time points may not have coincided with the peak detection of MPO protein.

Inflammation contributes to the pathogenesis of diabetes, in which SAA and other inflammatory markers, including CRP, TNF, and MCP-1, increase in parallel with urinary albumin (53). Serum SAA concentrations also correlate with the course of diabetes in human diabetic kidney disease and in murine models (59). Previous studies found increased production and deposition of SAA, particularly prominent within glomeruli and tubular interstitium typically colocalized with epithelial podocytes. Similar to results presented here, SAA increased NFκB activity in tubular epithelial cells and this was temporally associated with up-regulation of numerous chemokine and cytokine genes including MCP-1 (59). MCP-1 increases substantially in diabetic nephropathy and in addition to its chemokine activity can induce an NFκB-dependent fibrotic response in mesangial cells. Moreover, exogenous SAA promotes localized SAA production in mouse podocytes, consistent with the suggestion that SAA may be central to an amplification loop that potentiates kidney inflammation (59).

Here we show for the first time that SAA promotes renal oxidative stress and inflammation, reflected by lipid (membrane and polyunsaturated fatty acid) and protein (3-Cl-Tyr) oxidation, to a greater extent than ApoE^−/−^ mice treated with a low-level LPS or vehicle as controls. Lipid-mediated renal injury may also result from inflammation (60, 61). The lipid oxidation products CE-O(O)H and F_2_-isoprostanes increased in plasma and renal tissues 4 weeks after commencing SAA-treatment and remained higher elevated (above LPS or vehicle–treated mice) over 18 weeks. Lipid oxidation products can induce endothelial dysfunction via direct and indirect mechanisms (62). For example, F_2_-isoprostanes are vaso-constrictive in the renal and cardiovascular system and can cause platelet aggregation and smooth muscle proliferation (63). Similarly, CE-O(O)H can affect arterial vasoconstrictor responses (64) and stimulate EC monocyte adhesions (65). Consistent with the measurement of elevated oxidized lipids as a biomarker for renal oxidative stress in plasma and kidney tissues, antioxidant genes/proteins in the aorta and kidney were also increased, and likely represented a compensatory response to the increased oxidative stress seen in these organs early after SAA administration.

Endogenous antioxidant response genes GPx-1 and aortic and renal catalase, both involved in peroxide elimination were elevated in kidneys from SAA-treated mice. At least for GPx-1, elevated mRNA correlated with accumulation of GPx protein in renal tubules and paralleled increases in total GPx activity in the kidneys from mice treated with SAA. Increases renal GPx protein/activity are associated with renal protection (66). For example, peroxides can interfere with NO production and bioactivity and can impair endothelial function (62) and this may subsequently impact renal perfusion leading to renal ischemia. Increased catalase (as implied by the significant increase in CAT gene expression detected in aortae and kidneys from SAA-treated mice, Table 1) may also be interpreted as a response to moderate renal damage through enhanced consumption of hydrogen peroxide. Thus, the parallel increase in CAT and GPx implicates SAA in stimulating oxidative stress pathways in addition to its pro-inflammatory action.

Microalbuminuria, defined as urinary excretion of albumin at a rate of 30–300 mg/24 h (67) is a marker of endothelial dysfunction, vascular injury, and renal and cardiovascular disease, and is associated with increased risk for myocardial infarction. It is the earliest clinical sign of diabetic nephropathy and is predictive for chronic renal failure (68). In addition, CVD risk closely correlates with albuminuria, extending even to previously-defined, normal urinary albumin limits (54) and is an important risk factor in early CVD mortality (56, 57, 69). SAA stimulated an early increase in proteinuria, which persisted over 18 weeks, indicating sustained renal impairment following treatment with SAA.

Impaired NO bioavailability/bioactivity underpins endothelial dysfunction, which is an early step in the development of atherogenesis (62). Several explanations can account for diminished aortic cGMP under pro-inflammatory conditions:

- Notably, SAA adversely affects NO bioavailability in cultured EC through stimulating the formation of superoxide radical anions that can rapidly react with NO to yield the potent oxidant peroxynitrite (16), thereby subverting NO bioactivity. That the aortae from SAA-treated mice studies here showed diminished cGMP levels relative to the controls is consistent with SAA inhibiting NO bioactivity.
- Limitation of eNOS substrate bioavailability may explain the promotion of vascular oxidative stress and decreased vascular cGMP. However, we found that aortic arginase activity was not elevated in SAA-treated mice, that would otherwise implicate SAA in promoting *L*-Arg metabolism (to urea) and inhibiting NO biosynthesis by limiting eNOS substrate. However, plasma urea was increased in SAA-treated mice, likely reflecting altered rates of urea excretion resulting from SAA-induced kidney damage.
- This study also demonstrated an SAA-mediated increase in renal biomarkers of oxidative damage and NF-κB (NFκB *P*-P65) activation, which in combination can promote renal dysfunction by limiting NO bioavailability (16), which is completely consistent with the decreased levels of aortic cGMP detected here. SAA-stimulated NFκB activation enhances induction of pro-atherogenic factors that promote endothelial activation, manifested here by increased VCAM-1 expression in isolated aorta from ApoE^−/−^ mice treated with SAA.
- Paradoxically, NFκB activation is also linked with up-regulation of inducible NOS (iNOS) in cultured murine macrophages (69), which necessarily leads to an increase in NO production. Despite this indication, a decrease in aortic cGMP detected here would suggest that iNOS-mediated NO production under SAA-stimulated proinflammatory conditions is unable to activate sGC in vascular smooth muscle, possibly as a majority of this inducible NO pool is diverted from it biological target through reaction with oxidants that are produced simultaneously in the presence of SAA (16).

Taken together our data show that SAA likely affects NO bioactivity *in vivo* through multiple pathways; however, whether NO production is actually ameliorated or in fact increases through the up-regulation of the iNOS pathway remains to be tested.

HDL has intrinsic anti-inflammatory activity (70) and modulates pro-inflammatory/pro-thrombotic activities of SAA on EC (16). ApoE^−/−^ mice have lower levels of HDL than control mice (71) and this may be a limitation of this study. For example, it is feasible that the lipoprotein's capacity to limit SAA activity may be overwhelmed, thereby reducing its protective capacity. Alternatively, ApoE deficiency may impact on lipoprotein regulation of SAA activity. Therefore, further examination on the roles for HDL apolipoproteins in regulating SAA activities are warranted, particularly as HDL is considered protective in CVD.

## Conclusions and further limitations to the study

Our data show that SAA induces pro-inflammatory and pro-thrombotic activities, and oxidative stress in the absence of a high fat diet, promoting vascular and renal dysfunction. Renal dysfunction and CVD share common risk factors including diabetes, hypertension and dyslipidemia, all characterized by inflammation, oxidative stress, and resultant endothelial dysfunction similar to the outcomes identified here by introducing recombinant SAA into mice at a final dose of 10 μg/kg. At least in the case of endothelial dysfunction, this subtle pathology precedes clinical diagnosis of CVD and is a key contributor to cardiovascular and renal disorders. Pathways that underpin SAA-mediated vascular and renal dysfunction have implications for other inflammatory pathologies [e.g., cancer and rheumatoid arthritis (72)] where these altered parameters are common features in disease progression. We propose that increases in SAA may predispose to impaired renal function and increased cardiovascular risk, particularly in diseases that involve dyslipidemia and altered/low circulating HDL (73).

Several limitations complicate the interpretation of the data determined in this study:

- The biological activity of recombinant SAA has been questioned in a recent paper which suggests that contaminating bacterial lipopetides/lipoproteins constitute the pro-inflammatory action of recombinant SAA (74). Of note, a contemporary review of the field concludes that on balance commercially sourced recombinant SAA contains low-level LPS contamination (in our hands SAA preparations typically contained <2 pg detectable LPS/ μg of SAA) and the recombinant protein is able to elicit biological activity when judged against appropriately controlled studies (75).
- mice endogenously express isoforms SAA1, SAA2, and SAA3, which may exhibit differing bioactivity.

In this study we have gone to some length to detect contaminating LPS in SAA preparations and also employed LPS as a positive control in mice. Notably the sourced LPS (LPS-B5 derived from *E. coli* 055:B5) is not ultrapure (purified by gel-filtration) and therefore, contains other contaminating bacterial components, such as lipoproteins/peptides (76). Overall, the data presented here demonstrate that LPS, administered to aged-matched ApoE^−/−^ mice at final dose 25 pg/kg, slightly higher than the level estimated from contaminating levels in corresponding mice treated with recombinant SAA, yields a phenotype identical to the vehicle control group, that both differ significantly from mice administered recombinant SAA. Therefore, based on the comparison with vehicle and positive control groups, it appears that recombinant SAA is able to stimulate both vascular endothelial cell and renal dysfunction in mice. The precise mechanism for this bioactivity is unclear and whether endogenous SAA isoforms become altered by the presence of recombinant SAA and participate in this inflammatory response is unclear and warrants future investigation.

## Author Contributions

BC conducted the bulk of the experiments with AV and together produced a draft manuscript. FH conducted biochemical and molecular studies. TH conducted IHC, Western blotting, and selected gene studies. XC, RA, GF, AV, and NM conducted empirical studies. XW, BC, and AV conducted mass spectrometry. ArS, BC, AV, and WS conducted the leukocyte adhesion work. JD, JC-D, JdH, AlS, CG, BF, and PW conceptualized experiments, discussed research outcomes, and set research direction. This group was also responsible for developing the manuscript.

### Conflict of Interest Statement

The authors declare that the research was conducted in the absence of any commercial or financial relationships that could be construed as a potential conflict of interest.
