# Supplementary material for: Serum Amyloid A Stimulates Vascular and Renal Dysfunction in Apolipoprotein E-Deficient Mice Fed a Normal Chow Diet
Source: Front Immunol. 2019 Mar 7;10:380. doi: 10.3389/fimmu.2019.00380 (PMC6416175; doi:10.3389/fimmu.2019.00380)
Supplement: Supplementary file 1 [file Data_Sheet_1.pdf]

Serum amyloid A stimulates vascular and renal dysfunction in apolipoprotein E-deficient mice fed a normal chow diet

Belal Chami,[a] Farjaneh Hossain,[a] Thomas W. Hambly,[a] Xiaoping Cai,[a] Roshanak Aran,[a] Genevieve Fong,[a] Abigail Vellajo[a] Nathan J.J. Martin,[a] XiaoSuo Wang,[a] Joanne M. Dennis,[a] Arpeeta Sharma,[b] Waled A. Shihata,[c,d,e] Jaye P.F. Chin-Dusting,[c,d,e] Judy B. de Haan,[e] Alexandra Sharland,[f] Carolyn L. Geczy,[g] Ben Freedman,[h,i] and Paul K. Witting.[a,*]

[a]Discipline of Pathology, Sydney Medical School, Charles Perkins Centre, The University of Sydney, NSW, 2006 Australia; [b]Department of Medicine, Monash University, Victoria, Australia; [c]Cardiovascular Disease Program, Biomedicine Discovery Institute, Monash University; [d]Department of Pharmacology, Monash University, Victoria, Australia; [e]Baker Heart and Diabetes Institute, Victoria, Australia; [f]Transplantation Immunobiology Group, Central Clinical School, Sydney Medical School, Charles Perkins Centre, The University of Sydney, NSW, 2006; [g]School of Medical Sciences, University of New South Wales, Sydney 2052 Australia; [h]ANZAC Research Institute, and Heart Research Institute, Charles Perkins Centre, University of Sydney, Sydney, Australia; [i]Heart Research Institute, Charles Perkins Centre, The University of Sydney, NSW, 2006 Australia

*Correspondence to Associate Professor Paul Witting; Phone: 61-2-9114-0524, E-mail: paul.witting@sydney.edu.au

SUPPLEMENTAL MATERIAL

Supplementary Table I. List of antibodies and experimental conditions used for immuno-imaging.[a]

Fixed Tissues								
Antigen	**Primary Antibody**					**Secondary Antibody**		
	Company	Cat. No.	Clonality	Host Species	Dilution	Reactivity	Conjugated	Dilution
GPx-1	Abcam	ab59546	Polyclonal	Rabbit	1/250	Rabbit	Biotinylated	1/250
VCAM-1	Santa Cruz	sc-13160	Clone E-10	Mouse	1/500	Mouse	Biotinylated	1/250
Ly6G	Abcam	ab25024	RB6-8C5	Rat	1/500	Rabbit	IgG2b	1/250
TF	American Diagnostic	4509	Clone VD8	FITC Conjugated	1/500	N/A	N/A	N/A
Frozen Tissues								
**Primary Antibody**						**Secondary Antibody**		
	Company	Cat. No.	Clonality	Host Species	Dilution	Reactivity	Conjugated	Dilution
F4/80[b]	Walter & Eliza Hall	N/A	Polyclonal	Rat	1/200	Rat	Peroxidase; [c]Alexa Fluor 594	1/200
Myeloperoxidase	Abcam	ab9535	Polyclonal	Rabbit	1/50	Rabbit	FITC	1/200
NF-κB (phosphor-p65 ser276)	Signalway Antibody	11011	Polyclonal	Rabbit	1/50	Rabbit	FITC	1/200
Western blotting								
	**Primary Antibody**					**Secondary Antibody**		
	Company	Cat. No.	Clonality	Host Species	Dilution	Reactivity	Conjugated	Dilution
NF-κB (phosphor-p65 ser276)	Signalway Antibody	11011	Polyclonal	Rabbit	1/1000	Rabbit	Peroxidase	1/5000

[a] Commercial antibodies listed were obtained as undiluted stock solutions and diluted to the final concentrations indicated.

[b]We thank Associate Professor Bob Bao for providing a sample of this Antibody.

[c]Alexa Fluor conjugated antibody was used in NF-κB & macrophage (F4/80[b]) co-labelling IHC studies.

Table II. PCR primer sequences used for gene analyses.[a]

Gene	Sequence	Reference source/NCBI Reference Sequence
TF - F	TCAAGCACGGGAAAGAAAAC	1 / NM_010171.3
TF - R	CTGCTTCCTGGGCTATTTTG	
NF-κB p50 F	GGAGGCATGTTCGGTAGTGG	2 / XM_006501106.2
NF-κB p50 R	CCCTGCGTTGGATTTCGTG	
Gpx-1 F	TGAGAAGTGCGAGGTGAATG	3 / NM_008160.6
Gpx-1 R	AACACCGTCTGGACCTACCA	
CAT F	ACATGGTCTGGGACTTCTGG	4 / NM_009804.2
CAT R	CAAGTTTTTGATGCCCTGGT	
VCAM-1 F	ATGTCAACGTTGCCCCCAAG	NM_011693.3
VCAM-1 R	AATGCCGGAATCGTCCCTTT	
β-actin F	AGCCATGTACGTAGCCATCC	5-7 / NM_007393.5
β-actin R	CTCTCAGCTGTGGTGGTGAA	

Primers synthesized by Proligo (Sydney Australia) were diluted 10-fold to yield a working stock solution. Note, all PCR reactions were optimized to yield a single product band at the anticipated size based on BLAST search for the primer sequences employed here (accession numbers indicated). In addition for the assessment of TF, Gpx and VCAM-1 regulation, complementary assessment of protein expression was performed to validate the corresponding changes in gene regulation in the same renal tissue.

Figure S1.

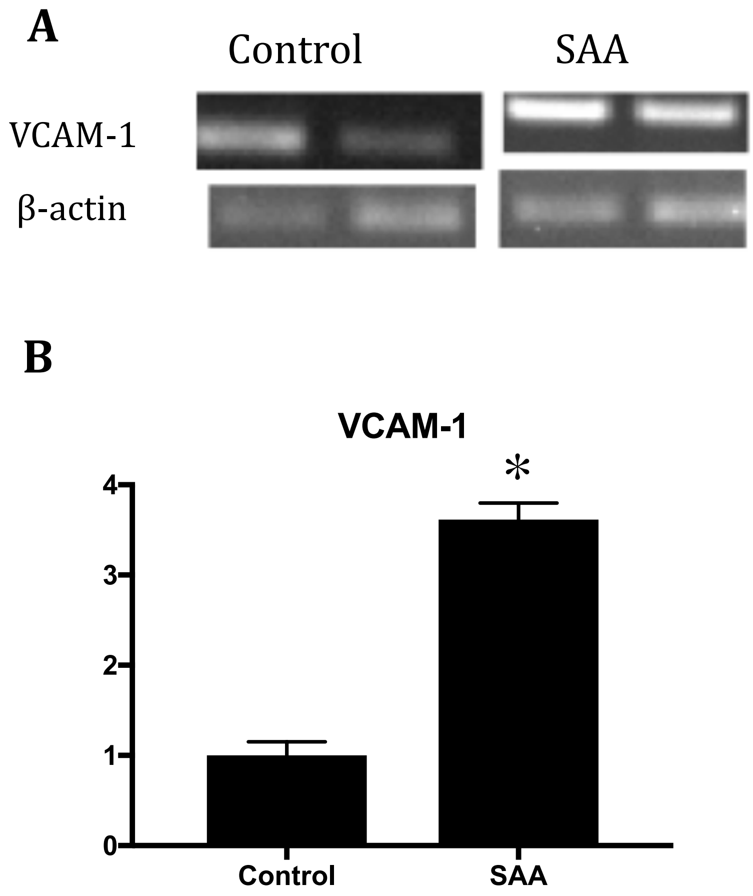

Supplementary Figure S1. Aortic VCAM-1 mRNA expression increased in Apo E-/- mice administered SAA for 2 weeks (as described in Study 2). as assessed by RT-PCR. Animals treated with vehicle (control) and SAA were sacrificed and thoracic aortae were carefully harvested and cleaned of fat to preserve vascular endothelium integrity. The cleaned aortic vessel was then mounted into a dynamic flow system and fluorescently labeled leukocytes were passaged into the vessels to assess leukocyte adherence to the vessel wall using real-time imaging available at the Baker Institute, Melbourne. Immediately after imaging the vessel segments were snap frozen in liquid nitrogen and transported to the Charles Perkins, Sydney. The samples were then homogenized, total RNA extracted and the corresponding cDNA probed for expression of VCAM-1 by RT-PCR and the outcomes finally expressed as a fold-change in density relative to the control (assigned an arbitrary value of 1). Images show (A) representative PCR products for housekeeping gene (β-actin) and VCAM-1 in two samples of aortic homogenate. Panel (B) shows the quantitative outcome from assessment of the product band densities using Image J software. Data represent mean ± SD; n=4 aorta per group, each analysis performed in triplicate. Different to vehicle-treated control *$P<0.05$.

Figure S2.

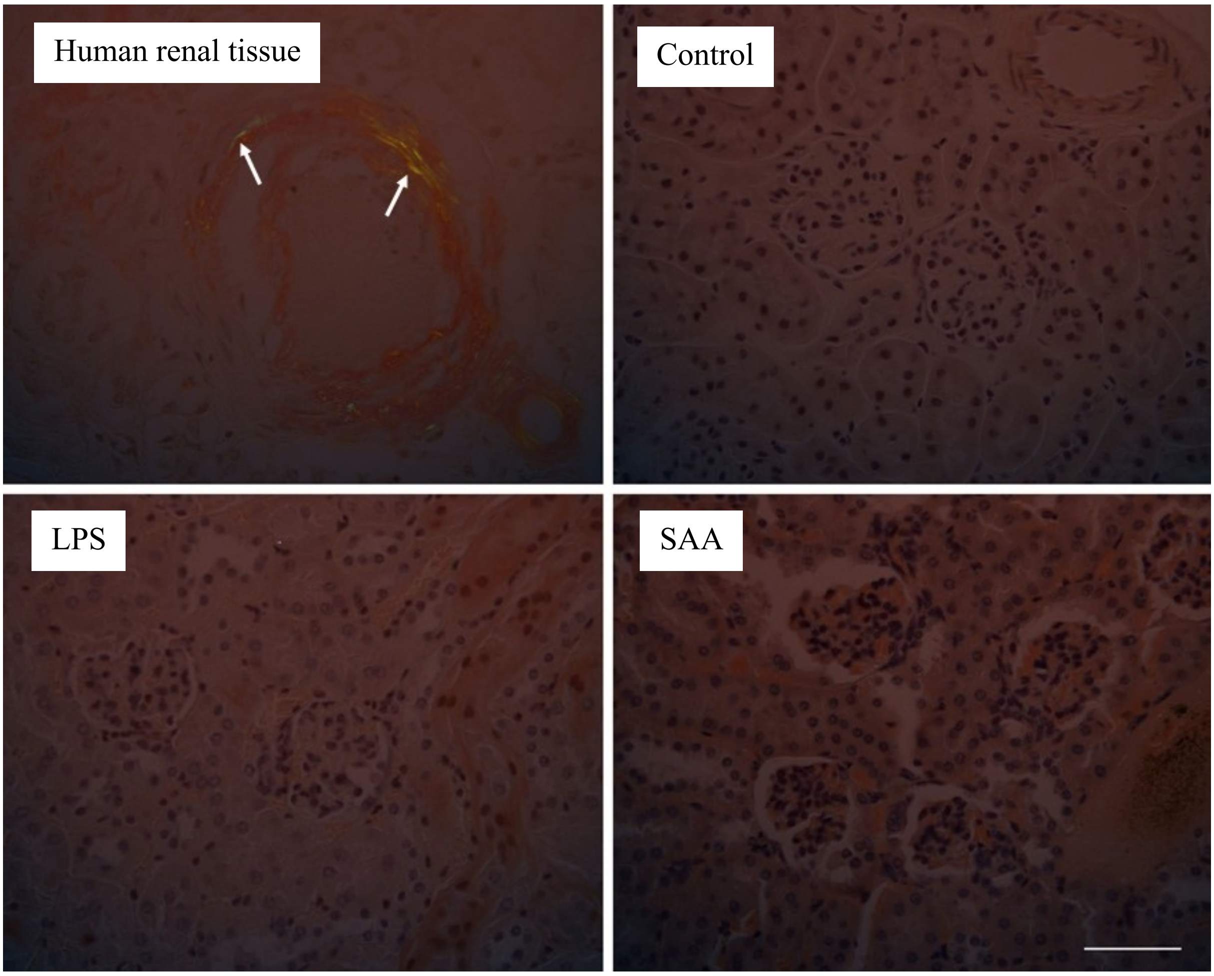

Supplementary Figure S2. Apo E-/- mice treated with SAA showed no sign of kidney amyloidosis. Animals treated with saline (control), LPS and SAA and 4 weeks after treatment commenced (as described in Study 1) and the mice were sacrificed and kidneys harvested. Renal tissues were embedded, sectioned, stained with Congo Red and imaged to visual birefringence (representing cortical amyloidosis) under polarized light. Representative sections shown are obtained from positive control showing amyloidosis as yellow birefringence (white arrows indicates yellow and green birefringence). Note, human renal tissue (positive control) was obtained from the Discipline of Pathology; University of Sydney), and mouse kidney sections from control, LPS and SAA-treated animals. No meaningful birefringence was detected in sections from mouse kidneys. Figures are representative of at least three independent samples from each treatment group. All images were captured at x400 magnification. Arrow indicates amyloidosis. Scale bar= 50µM.

Figure S3.

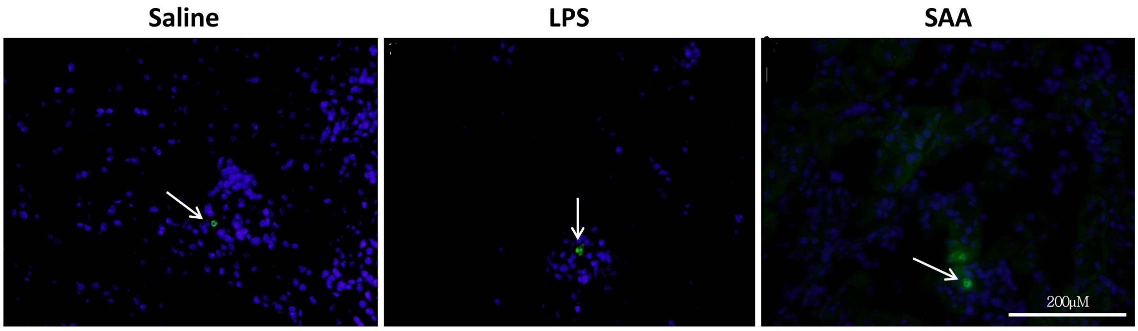

Supplementary Figure S3. **Immune-histochemical labelling of Ly6G$^+$ neutrophils in kidneys from Apo E-/- mice.** Mice were treated daily with sterile saline (vehicle), LPS or SAA for 3 days as described in the *Materials and Methods* section prior to kidney excision. Neutrophils (arrows) were labelled with a FITC conjugated rat anti-mouse Ly6G antibody (green), and nuclei stained with DAPI (blue). Images were taken at Magnification x200; scale bar, 200 µm. Complementary assessment of frequencies of Ly6G$^+$ cells in kidneys isolated from mice 5 days after treatment with vehicle, LPS or SAA confirmed the absence of infiltrating neutrophils at that time (data not shown).

Figure S4.

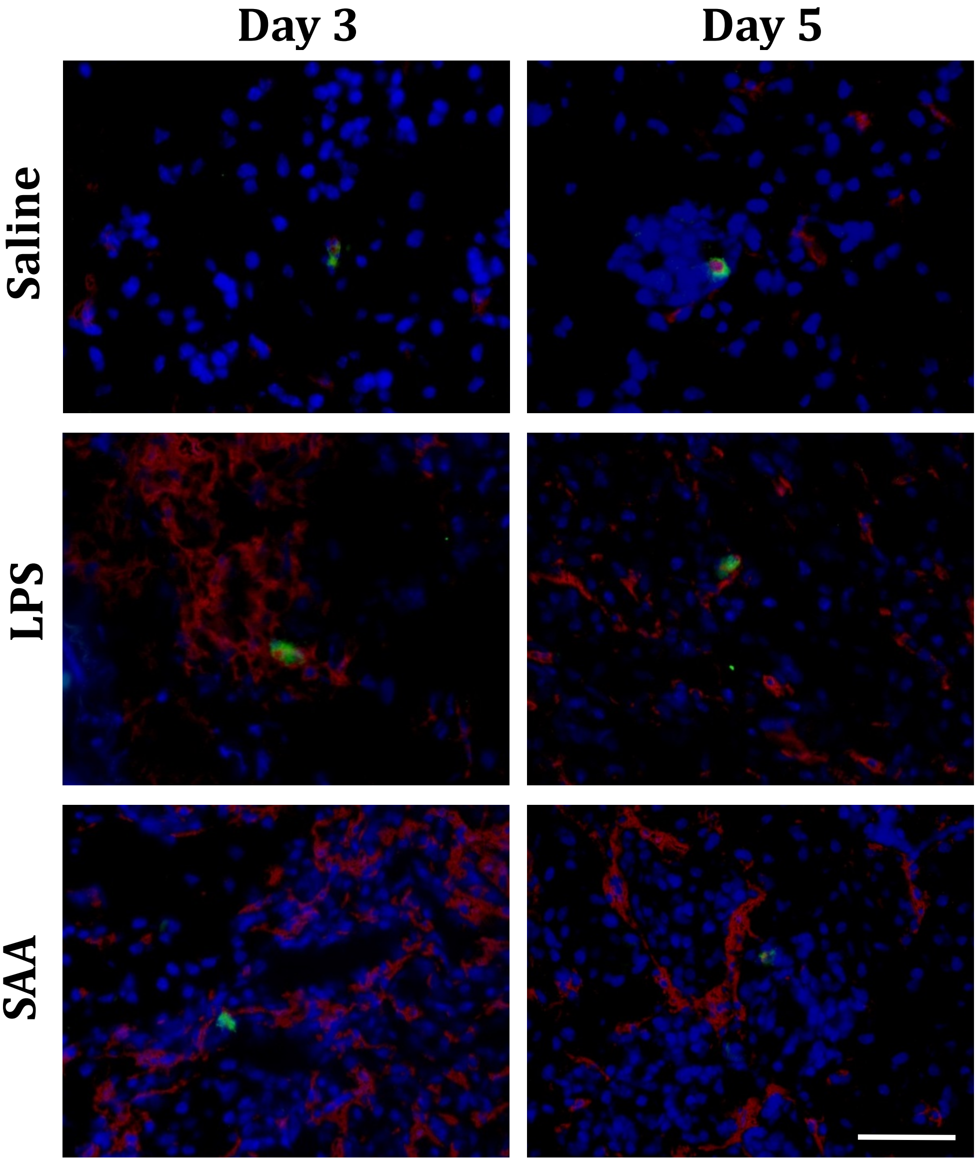

Supplementary Figure S4. Identification of macrophages expressing myeloperoxidase protein in kidneys from SAA-treated mice. Mice were treated with sterile saline (vehicle), LPS or SAA as described in the *Materials and Methods* section. Kidneys were isolated after 3 or 5 days post commencement of SAA treatment and the frozen kidneys were sectioned and immune-stained for F4/80 and MPO proteins. Images were taken to highlight F4/80$^+$ macrophages with little evidence of MPO$^+$ immune-staining. Overall, there were no obvious differences in MPO$^+$ immune-labelling in any of the treatment groups. Here, nuclei are stained with DAPI (blue), F4/80 (a murine macrophage marker) is stained with an HRP/DAB system (processed to appear red) and myeloperoxidase is labelled with FITC (green). Magnification, x 200; scale bar 100 μM.

Figure S5.

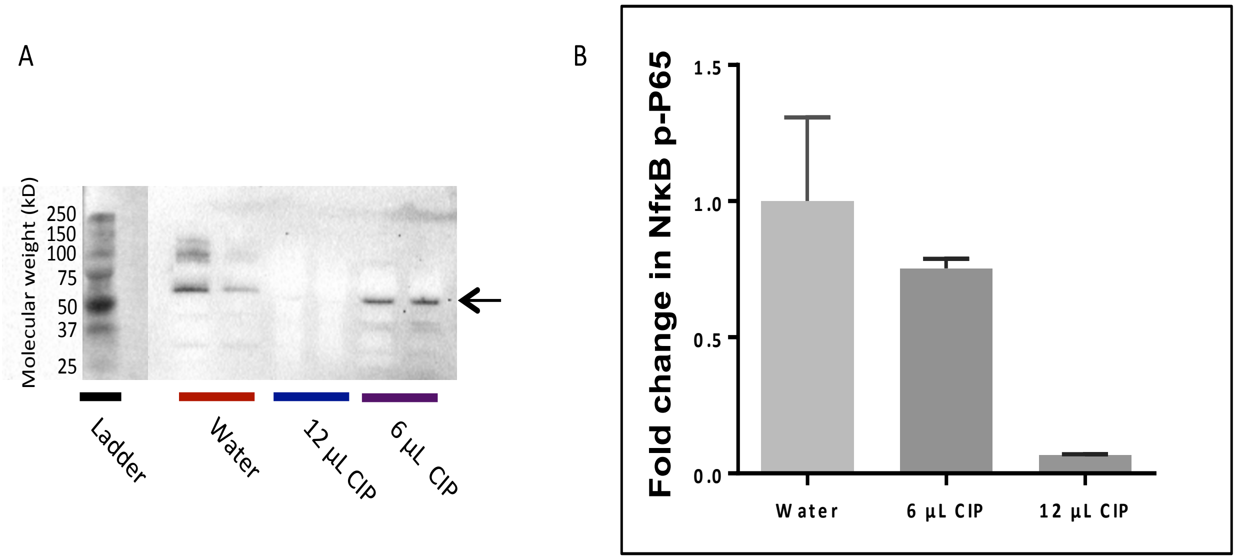

Supplementary Figure S5. Confirmation of NF-κB *P*-P65 assignment by use of a phosphatase to dephosphorylate *P*-P65. Kidney homogenates expressing NF-κB *P*-P65 (phosphorylated at ser276; 60 kDa) were incubated with water (control), or buffer containing 6 or 12 µL calf intestinal phosphatase as described in the *Methods Section* in the main paper. Samples were then assessed for NF-κB *P*-P65 by Western blotting (refer to Methods for details). Representative blot shows (A) protein bands of interest (see arrow) that were (B) quantified and normalised against total protein values by densitometry analysis of UV images of the total protein bands detected in-gel. Panel (B) shows mean fold changes relative to water-treated samples ± maximal error; n=2 independent experiments.

Supplementary references

1. Chantrathammachart P, Mackman N, Sparkenbaugh E, Wang J-G, Parise LV, Kirchhofer D, Key NS, and Pawlinski R: Tissue factor promotes activation of coagulation and inflammation in a mouse model of sickle cell disease. Blood. 2012, 120: 636–46.

2. Ross MJ, Martinka S,D'Agati VD, Bruggeman LA: NF-κB regulates Fas-mediated apoptosis in HIV-associated nephropathy. Journal of American Society of Nephrology. 2005, 16: 2403–11.

3. Chang CY1, Song MJ, Jeon SB, Yoon HJ, Lee DK, Kim IH, Suk K, Choi DK, Park EJ. Dual functionality of myeloperoxidase in rotenone-exposed brain-resident immune cells. Am J Pathol. 2011 Aug;179(2):964-79.

4. Cui Y, Wang Q, Li X, Zhang X: Experimental nonalcoholic fatty liver disease in mice leads to cytochrome p450 2a5 upregulation through nuclear factor erythroid 2-like 2 translocation. Redox Biology, 2013, 1:433–40.

5. Suliman HB, Welty-Wolf KE, Carraway MS, Schwartz DA, Hollingsworth JW, Piantadosi CA. Toll-like receptor 4 mediates mitochondrial DNA damage and biogenic responses after heat-inactivated E. coli. FASEB Journal 2005, 19:1531-3.

6. Mork CN, Faller DV, Spanjaard RA. Loss of putative tumor suppressor EI24/PIG8 confers resistance to etoposide. FEBS Lett. 2007, 581:5440-4.

7. Shim YM1, Zhu Z, Zheng T, Lee CG, Homer RJ, Ma B, Elias JA. Role of 5-lipoxygenase in IL-13-induced pulmonary inflammation and remodeling. Journal of Immunology. 2006, 177:1918-24.
